# Photo enhanced degradation of polyfluoroalkyl and perfluoroalkyl substances

**DOI:** 10.1016/j.heliyon.2020.e05614

**Published:** 2020-12-01

**Authors:** Olalekan C. Olatunde, Alex T. Kuvarega, Damian C. Onwudiwe

**Affiliations:** a Material Science Innovation and Modelling (MaSIM) Research Focus Area, Faculty of Natural and Agricultural Sciences, North-West University, Mafikeng Campus, Private Bag X2046, Mmabatho 2735, South Africa; b Department of Chemistry, School of Physical and Chemical Sciences, Faculty of Natural and Agricultural Sciences, North-West University, Mafikeng Campus, Private Bag X2046, Mmabatho 2735, South Africa; c Nanotechnology and Water Sustainability Research Unit, College of Science, Engineering and Technology, University of South Africa, Florida 1709, South Africa

**Keywords:** Photodegradation, Perfluorinated compounds, Photolysis, Defluorination, Photoreduction

## Abstract

The increase in the presence of highly recalcitrant poly- and per- fluoroalkyl substances (PFAS) in the environment, plant tissues and animals continues to pose serious health concerns. Several treatment methods such as physical, biological and chemical processes have been explored to deal with these compounds. Current trends have shown that the destructive treatment processes, which offer degradation and mineralization of PFASs, are the most desirable process among researchers and policy makers. This article, therefore, reviews the degradation and defluorination processes, their efficiencies and the degradation mechanism of photon-based processes. It shows that high degradation and defluorination efficiency of PFASs could be achieved by photon driven processes such as photolysis, photochemical, photocatalysis and photoreduction. The efficiency of these processes is greatly influenced by the nature of light and the reactive radical generated in the system. The limitation of these processes, however, include the long reaction time required and the use of anoxic reaction conditions, which are not obtainable at ambient conditions.

## 1 Introduction

**Figure 1:**
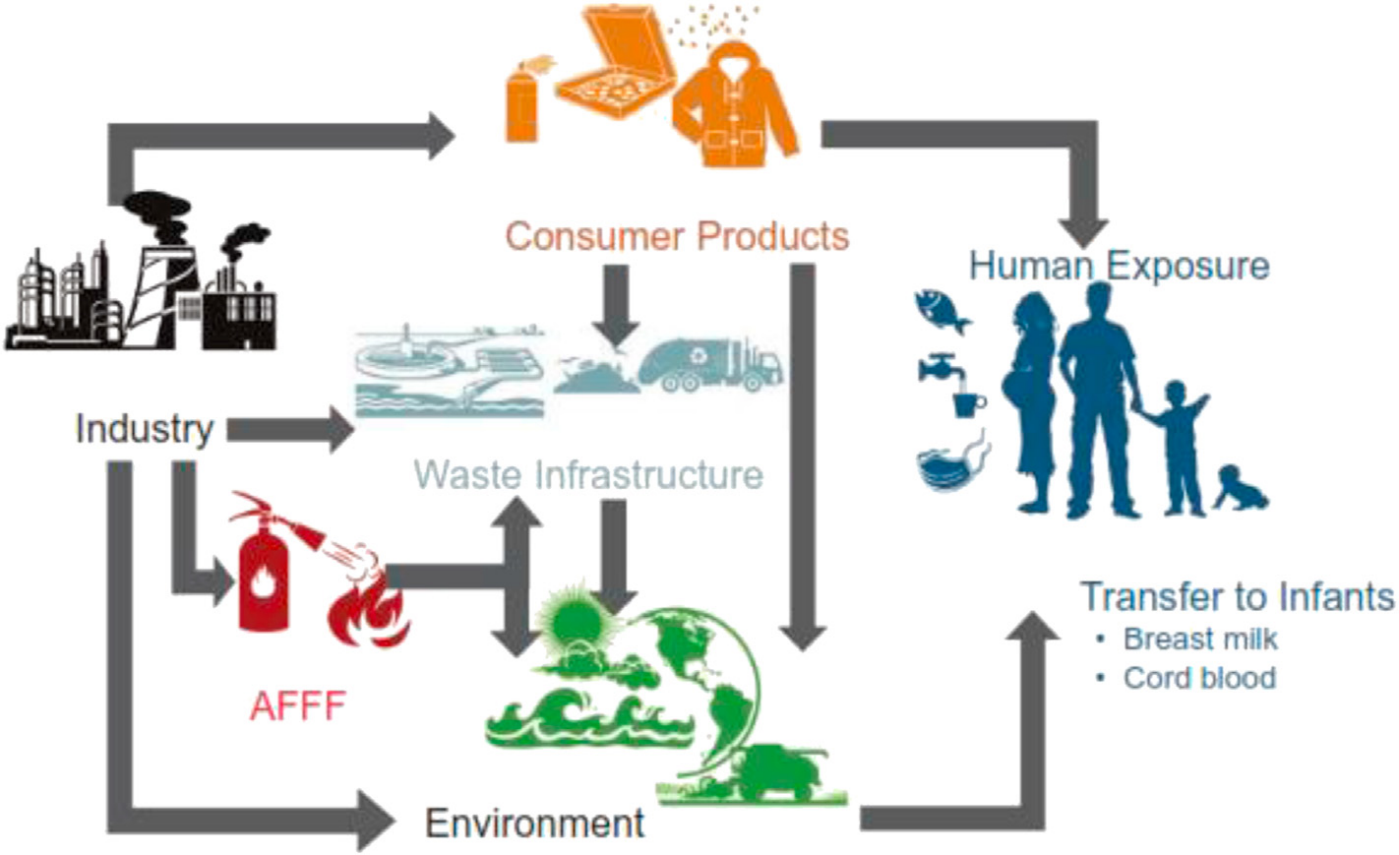
Exposure pathways to PFAS. Reproduced with permission from Sunderland *et al.* [6]. Copyright (2020) Springer Nature.

Polyfluoroalkyl and perfluoroalkyl substances (PFASs) are class of highly fluorinated aliphatic substances. They contain one or more carbon atoms in which the hydrogen atoms present in their non-fluorinated analogues, from which they have been supposedly derived, have been replaced by fluorine atoms, in such a way that they contain the perfluoroalkyl moiety C_n_F_2n+1_^–^ [1]. The public health attention on PFAS is due to their persistence, capacity for long range transport in the environment, potential bioaccumulation and adverse effect on living organisms [2, 3]. The source of PFAS could either be direct or indirect, and their release into the environment occurs throughout their life cycle (i.e. production, distribution, use, and disposal of consumer products) [4]. While direct sources are the releases from the life cycle of PFAS products, indirect sources are transformation products of precursor substances and impurities from consumer products [1]. The various exposure pathways for PFAS include drinking water, food-contact materials, foods, airborne dust, air, breast milk etc (Figure 1) [5].

Studies on the toxicological effects of PFAS exposure using laboratory animals as model showed the potential health effects to include: tumour induction, immunotoxicity, hepatotoxicity, endocrine disruption, developmental toxicity and neurotoxicity [7]. In humans, PFAS have been reported to bioaccumulate in the blood even at trace level of exposure and correlations between exposure to PFAS and disease parameters immunotoxicity have been demonstrated experimentally [8, 9]. These concern over the potential environmental and toxicological effects of PFAS have led to environmental agency such as the US Environmental Protection Agency (USEPA) to enter into agreements with some leading global companies in order to reduce the emission and product content of perfluoro octanoic acid (PFOA) and related chemicals up to 95% by 2010 and aim at their complete elimination by 2015 [10]. In line with this also, in May, 2020, Demark published an executive order which prohibits the use of PFAS chemicals in food contact paper, board materials and items [11].

PFAS are widely distributed in marine, ground, surface and drinking waters at concentration levels of pg/L to μg/L. Close proximity of an aquatic system to industrial sources could increase the concentration of PFASs by several order of magnitude [12]. In some wastewater, the level of PFASs could be in the range of mg/L to g/L. For example, the concentration of perfluorooctanoate sulfonate in wastewater from one of the electronics manufacturing companies was found to be 1,650 mg/L, which was 10 billion times more than the concentration level in the open oceans [13]. The distribution of PFAS in the environment is influenced by their physicochemical properties which include carbon chain length, solubility in water, volatility and headgroup functionality [14]. The physicochemical properties of some commonly studied PFAS are shown in Table 1. Short-chain PFAS have been proposed as safer substitutes for long-chain PFAS due to their shorter half-lives in human, however little research is available on their toxic effects based on persistence, exposure and retention [15]. Two long chain PFAS that have been studied extensively in remediation studies are perfluorooctanoic acid (PFOA) and perfluorooctane sulfonate (PFOS).

**Table 1:** Physicochemical properties of some PFASs.

Group	Name	Molecular formula	Molecular weight (g/mol)	Solubility^a^ (g/L)	PKa^a^	Ref.
Perfluoroalkyl sulfonic acids (PFSA)	Perfluorobutanesulfonic acid (PFBS)	C_4_HF_9_SO_3_	300.1	46.2	0.1	[16, 17]
	Perfluorohexanesulfonic acid (PFHxS)	C_6_HF_13_SO_3_	400.1	0.13	0.14	
	Perfluorooctane sulfonic acid (PFOS)	C_8_H_17_SO_3_	500.1	0.57	-3.3	
Perfluoroalkyl carboxylate (PFCA)	Perfluorobutanoic acid (PFBA)	C_4_HF_7_O_2_	214.0	1.5	-	
	Perfluorohexaonic acid (PFHxA)	C_6_HF_11_O_2_	314.1	0.1	-0.16	
	Perfluorooctanoic acid (PFOA)	C_8_HF_15_O_2_	414.0	3.4	-0.2	

a Obtained from PubChem Compound Summary for CID 67734, Perfluorohexanesulfonic acid 2020 [Available from: https://pubchem.ncbi.nlm.nih.gov/compound/Perfluorohexanesulfonic-acid [18].

In the last two decades, studies have focused on the removal of PFAS from water, and the results have shown that PFASs are posing serious risk to water resources and challenging traditional practices such as environmental discharges and recycling. Because typical wastewater treatment systems are unable to remove PFASs, they can serve as sinks for them and act as conduits for accessing the environment through effluent discharge and biosolid applications [19]. A study to investigate PFASs concentration and removal in three wastewater treatment systems, showed the level in influent, effluent and dry weight sludge to be 19.6–232 ng/L, 15.5–234 ng/L and 31.5–49.1 ng/g dry weight respectively. While PFASs were not eliminated effectively by the conventional activated sludge system, membrane bio-reactor and Unitank could achieved 50% removal of long chain perfluorocarboxylic acids (PFCAs) [20].

Despite the huge challenge posed by PFAS to water resource management and the potential adverse health implications, their use has continued to grow due to their economic importance evidenced by their application in a wide range of industrial, commercial and residential applications such as stain repellents, paints and coatings, polishes and surfactants in paper, metal plating, textile and household articles [21, 22, 23]. It has, therefore, become important to develop processes with sufficient capability to deal with PFAS in water and also up-scale the processes as a matter of global emergency [24].

Remediation processes employed in the removal of contaminants from water could be broadly classified as either destructive or non-destructive as shown in Table 2 [25]. Non-destructive processes usually involve the physical transfer of contaminants from an aqueous phase to a solid phase. It comprises of physical removal processes such as adsorption and membrane filtration [26, 27, 28]. Although, high removal efficiency of PFAS have been achieved with non-destructive techniques, the waste generated by these processes such as spent adsorbent and membranes are usually employed as landfills. This give rise to the possibility of re-contamination as these contaminants could leach back into the environment. These waste streams therefore, require further processing which could lead to a further increase in the process cost.

**Table 2:** Comparison of remediation methods for the removal of PFAS.

Class		Types	Mechanism	Main component	Advantage(s)	Disadvantage(s)	Ref.
Non-Destructive techniques		Adsorption	Electrostatic interaction between the polar groups on adsorbents and charged functional groups on PFAS or hydrophobic interaction between apolar adsorbents and non-polar tail of PFAS.	Adsorbent	Use of low-cost adsorbent materials and low energy demand.	Lack of sorbents with molecular specificity and affinity for PFAS. This process is also significantly influence by background water chemistry. Frequent regeneration of PFAS laden adsorbent is required due to fast breakthrough and disposal of PFAS concentrate is also a major challenge.	[42, 43]
		Membrane technology	Removal based on charge and size exclusion or by sorption	Membrane	High removal efficiency for both short and long chain PFAS. Minimal fouling	High energy demand and lack of selectivity. Waste streams that require further processing are generated	[44, 45]
Destructive techniques	Oxidative degradation	Electrochemical process	Degradation of PFAS based on the generation of reactive oxygen species ^•^OH by splitting of water or by direct anodic oxidation	Anode electrode e.g. B-doped diamonds, Ti/SnO_2_-Sb	Rapid reaction rate, high oxidation efficiency and ease of automation.	Toxic chlorine gas, HF, bromate, adsorbable organic halides and perchlorate are generated in the process. Potential generation of hazardous materials by the cavitation process. Large scale application is limited by several factors such as poor heat dissipation, transducer corrosion, required operation frequency and energy consumption. Rate of degradation is slow and thus usually require long reaction time and process efficiency is greatly influence by process parameters such as pH and presence of natural organic matters. Large chemical dosage may affect the cost effectiveness of the process Feasibility for treating large volume of contaminated water is less practical due to limited light penetration, high energy cost and difficulty in catalyst recycling and reuse.	[41, 46]
		Sonochemical process	Degradation is achieved by reactive species such as ^•^OH, H atom, O atom and $e_{aq}^{-}$ generated by acoustic cavitation process along with high temperatures and pressures	Transducer and amplifier	It's a highly clean, safe and energy saving process. No secondary pollution is caused.		[38, 47, 48]
		Chemical/Photochemical processes	Reactive chemical species such as ^•^OH, $O_{2}^{•\;-}$, $HO_{2}^{\;-}$ and $SO_{4}^{•\;-}$ are generated from oxidants like H_2_O_2_ and $S_{2}O_{8}^{2-}$. The generation of the reactive species could be enhanced by the use of photon energy for photochemical processes.	Light source and oxidant.	Mild reaction conditions, ease of automation and relatively cost-effective processes.		[36, 49]
		Photocatalytic process	Charge carriers generated from semiconductor photocatalyst when irradiated with light of appropriate energy reacts with oxidants to produce reactive radicals.	Light source and catalyst	Easy of incorporation with other technologies and no generation of secondary waste stream.		[50]
		Photolysis	Degradation is accomplished by the absorption of UV radiation <200 nm. Other radical species like $e_{aq}^{-}$ could also be generated in the process	Radiation source.	It is a green process with no addition of chemicals required.	Defluorination is generally low in photolytic process and processes depends on the absorptivity of the pollutant in the radiation range.	[51, 52, 53]
		Radiolytic process	Reactive radicals such as ^•^OH, ^•^H and $e_{aq}^{-}$ are obtained by water radiolysis, which then degrades dissolved pollutants.	Radiation source	The process may be a considered a green technology because it does not involve the use of any chemical. High efficiency in OH and $e_{aq}^{-}$ generation. Radiation generation technology are widely available.	High cost, limited knowledge about safety and continuous emission of rays sometimes result in permanent decrease in activity with time	[54]
	Reductive degradation	Reduction processes	Activation of sulphite, dithionite, ferrocyanide and aqueous iodide, by energy sources like ultrasound, electron beam or UV generates highly reactive, and nonselective reducing species such as H-, $S_{\;}O_{3}^{-}$ and $e_{aq}^{-}$	Energy source and reductant	Effective degradation of a wide range of PFAS	The need for pH adjustment and anoxic system conditions makes system operation, design and maintenance complex. Potential formation of undesirable degradation byproducts are also a main concern	[55]

Destructive remediation processes involve the degradation of contaminants to less toxic, and biodegradable products. For PFAS degradation, the process follows the cleavage of the C-F bond. This is achieved by the use of reactive radicals which are generated either via sonochemical, electrochemical, or photon-based processes [29, 30, 31, 32]. The photon-based processes are preferred to other processes, because they could be carried out at ambient temperature and pressure, and the process could be conducted in very simple reaction systems [33, 34, 35]. Depending on the components, a photon-based process can be classified either as photolysis, photochemical reaction or photocatalysis. In addition, based on the dominant reactive radical species generated, photon degradation process could either take place via oxidation or reduction [36, 37, 38]. Processes that occur via oxidation are referred to as advanced oxidation processes (AOP), while processes that occur via reduction are referred to advanced reduction processes (ARP). These two processes are at the fore front for PFAS degradation.

There are several reviews that have been written on the degradation of PFAS in water [38, 39, 40, 41], however, this review will focus mainly on photon-based degradation processes and also explore a comparative analysis of the different photon-based processes. The aim of this review is to therefore, critically access the level of efficiency that have been attained in the degradation of PFAS through the use of photon energy in both AOP and ARP processes. Particular interest will be on the types of light sources, oxidants and catalysts used. The mechanism of the degradation process will also be explored in order to have an understanding of the route to degradation product generation. A comparative analysis of the different photon-based processes will also be explored.

## 2 Classifications and properties of PFASs

**Figure 2:**
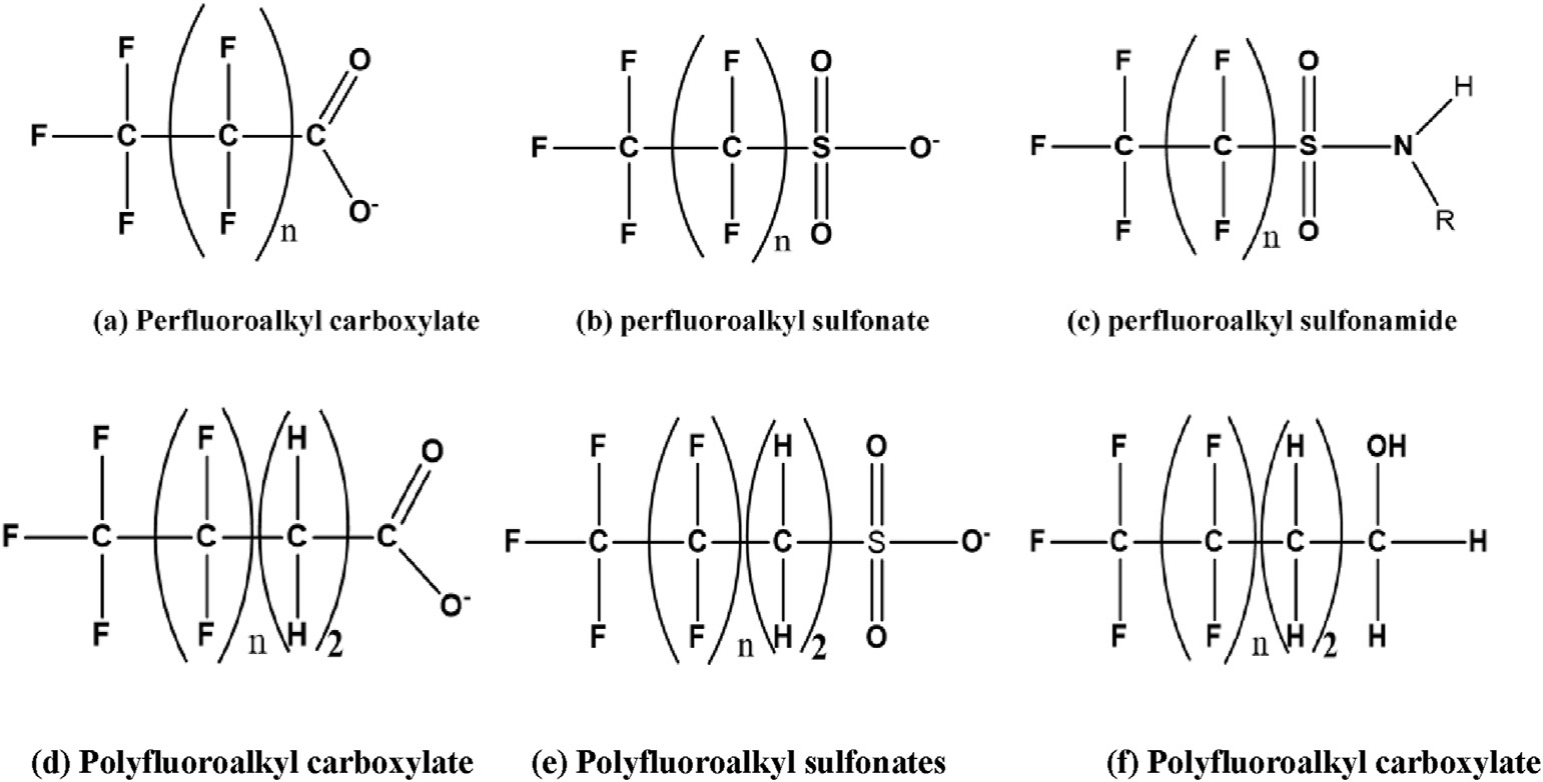
Structural formula of some perfluoro and polyfluoroalkyl substances.

A large group of industrial chemicals comprising of over 4700 chemicals are categorized as PFAS [56]. These chemicals could be further divided into three different classes: perfluoroalkyl substances, polyfluoroalkyl substances and fluorinated polymers. The perfluoroalkyl class is comprised of substances such as: perfluoroalkyl sulfonates (PFSAs), perfluoroalkyl carboxylates (PFCAs), perfluoroalkyl phosphonates (PFPAs), perfluoroalkyl sulfonamides (FASAs) etc. The main distinguishing structural characteristics of perfluoroalkyl substances is that they possess fully fluorinated alkyl chain unlike polyfluoroalkyl substances, which are only partially fluorinated as shown in Figure 2. Compounds grouped under the polyfluoroalkyl substances class include polyfluoroalkyl phosphoric acid esters (PAPs), fluorotelomer carboxylates (FTCA), fluorotelomer sulphonates (FTSAs) etc. The fluorinated polymers are a wide variety of chemicals that are further subdivided into: fluoropolymers, side-chain fluorinated polymers and perfluoropolyesters [57].

PFASs can also be classified based on their carbon length carbon chain length. as either long-chain or short chain. PFCAs with eight carbons or more and PFSAs with six carbons or more are classified as “long-chain” [1].

**Figure 3:**
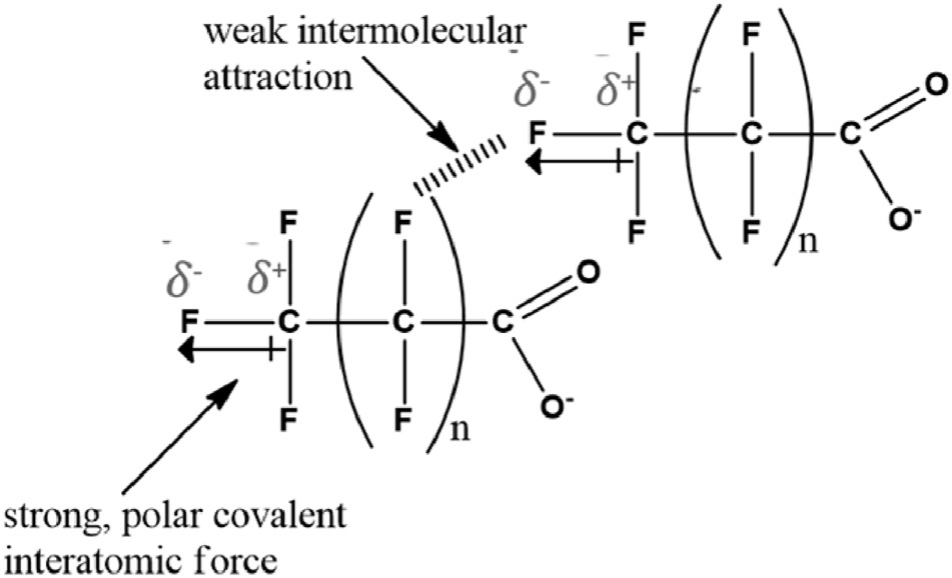
Interatomic and intermolecular interactions in perfluoroalkyl carboxylate.

PFASs possess some important structural properties such as long carbon chain length, functional groups and hydrophilicity/hydrophobicity, which influence their distribution and persistence in the environment. Due to the high electronegativity and the small atomic size of fluorine, a highly polar C-F bond with large bond energy is formed in PFASs as shown in Figure 3 [4]. This strong bond coupled with the presence of three nonbonding electron pairs on each fluorine atom and the effective shielding of the carbon atoms by fluorine confer relative stability on PFAS. Therefore, PFAS are resistant to many degradation processes involving acids, bases, reductants, oxidants, microbes, photolytic and metabolic process and they are also non-flammable [4, 58].

## 3 Advanced oxidation processes for PFAS degradation

### 3.1 Photolysis

Photolysis involves the direct use of photon for the degradation process. It usually employs ultraviolet (UV) radiations as most chromophores absorbs in this region. The UV light employed in photolysis can be classified into three wavelength ranges: UVA (315–400 nm), UVB (280–315 nm) and UVC (100–289 nm). Generally, wavelengths below 200 nm are referred to as vacuum UV (VUV) [59].

Due to the strong C-F bond, photolysis usually shows low efficiency for most PFAS degradation [52]. However, few studies have shown that effective photolysis of PFOA can be achieved by VUV light because the compound shows strong absorption in this region [51]. Giri *et al.* [60] reported the photolysis of PFOA, with 184 nm VUV light source and initial PFOA concentration of 2.42 × 10^−6^ mol/L. The degradation efficiency of the process reached 87.3% after 3 h, while 20.5% defluorination was achieved. The degradation and defluorination rate for the process were 0.82 and 0.143 h^−1^ respectively. Chen *et al.* [51], also reported a 61.7% degradation efficiency and 17.1% mineralization of 41.4 mg/L of PFOA after 2 h, using a 185 nm VUV light. The effect of combined UV and VUV wavelength (185 nm + 254 nm) on the photodegradation of PFOA has been investigated by Giri *et al.* [52]. The degradation process was significantly improved by combining the UV wavelength with VUV light, with complete degradation of 0.5 mg/L achieved after 4 h, while 254 nm UV light only achieved approximately 33% degradation. Kinetic studies on the process showed that the first-order rate constant for the combined wavelength process was 4.0 folds of the rate constant obtained for the single wavelength process. The percentage defluorination achieved by the combined wavelength process after 4 h was approximately 16%, while only 0.7% defluorination was recorded for the single wavelength process.

Evaluation of the effect of five process parameters: UV wavelength, dissolved oxygen, pH, PFOA concentration and water quality on efficiency of PFOA degradation was reported by Giri *et al.* [60]. While the nature of light source influences the rate of ROS generation, the pH influences the speciation of PFOA in solution which could also affect the efficiency of degradation. The water quality is a measure of parameters such as total alkalinity, dissolved oxygen, total dissolved solid etc, and often leads to the generation of several radical species or interact with radical species generated in the system. Dissolved oxygen may influence the activity of ROS and PFOA initial concentration also could influence the efficiency of the process by its light absorption capacity. The authors observed that PFOA photolysis was not significantly influenced by the initial concentration of the compound, though, a reduction in defluorination efficiency was observed with increase in the initial concentration of PFOA. The impact of pH on the degradation system also showed a negligible small effect on the degradation process. Dissolved oxygen negatively impacted the degradation efficiency of the process, but relatively better mineralization was achieved at low temperature values. The highest impact on efficiency was recorded by the effect of water quality, with organic constituents and bicarbonates, drastically reducing the photo-mineralization efficiency of the process [60].

### 3.2 Photochemical processes

Photochemical photon-based processes involve the combination of a light source with an oxidant, which undergoes photolysis to generate reactive radical species that attacks contaminants leading to their degradation. When the radical generated in the process is an oxygenated specie, the process is referred to as reactive oxygen species (ROS) based process. Oxidants employed in ROS processes include hydrogen peroxide, (UV/H_2_O_2_), persulfate (UV/$S_{2}O_{8}^{2-}$), ozone (UV/O_3_) and peroxymonosulfate ($UV/HSO_{5}^{-}$). A process that generates chlorinated species are called reactive chlorine (RCS) based processes (UV/Cl_2_). Recently, reactive nitrogen specie (RNS) based processes have been explored for the degradation of contaminants.

Photochemical processes that produces ^•^OH alone are not effective for PFAS degradation; and this is because perfluorinated compounds do not possess preferred reactive sites for reaction with the radical. Due to the lower dissociation energy of the F-OH bond compared to C-F bond, fluorine abstraction is not thermodynamically favoured [61]. Also, electron transfer reaction is hindered due to the reduction of the electron density of the ionic head group by perfluorination [62]. It is therefore, important for ^•^OH to be coupled with other radicals such as $e_{aq}^{-}$, which can interact with PFAS to form intermediates that are capable of undergoing addition reaction with ^•^OH [63]. To improve the efficiency of photochemical degradation of PFAS, a number of other oxidants have been explored such as persulfate, ozone and chlorine.

(1) $$S_{2}O_{8}^{2-}+hv\to 2SO_{4}^{•\;-}$$

(2) SO4•−+H2O→SO42−+O•H+H+

A number of reports have shown that persulfate is an effective oxidant in the degradation of PFAS [64, 65, 66]. In this process, degradation is achieved by $SO_{4}^{•\;-}$ and O•H generated by the activation of persulfate either by heat, light or chemical activation [65]. In a photochemical degradation system, the reactive radical generation is by Eqs. (1) and (2) [67]. $SO_{4}^{•\;-}$ is a very reactive radical with a redox potential of 2.6–3.1 eV. Its comparative stability and pH independent activity, makes it reactive with a wide range of pollutants [67]. It also react with electron-rich moieties via various reaction routes such as hydrogen abstraction, electron exchange and direct oxygen transfer [68]. Hori *et al.* [69] reported the complete degradation of 1.35 mM PFOA by 50 mM $S_{2}O_{8}^{2-}$ irradiated with a 200 W xenon-mercury lamp for 4 h.

**Figure 4:**
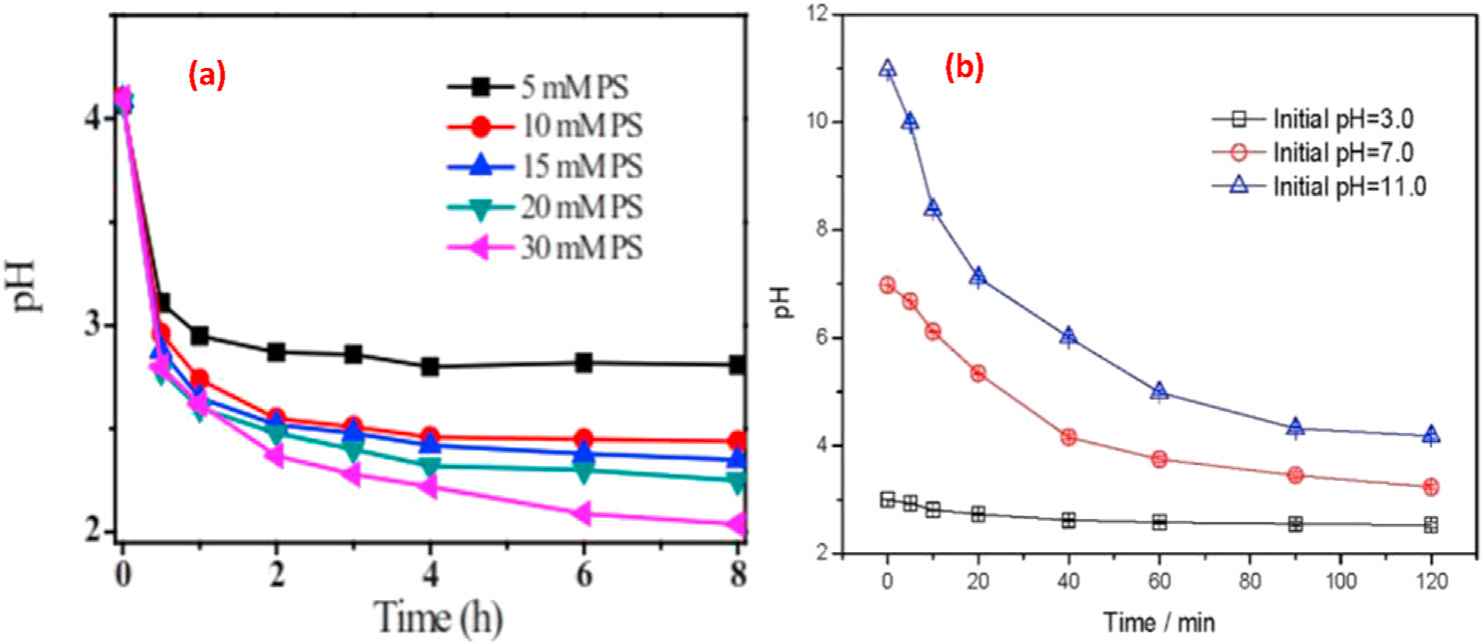
pH variation in a UV/persulfate process (a) pH variation with time for different initial concentration of PFOA. Reproduced with permission from Qian *et al.* [72]. Copyright (2016), American Chemical Society (b) pH variation with time for different initial system pH reproduced from Yang *et al.* [68] under the creative common attrition licence.

Studies have confirmed that the pH of the system in a UV/persulfate changes due to the generation of proton during persulfate activation [70]. This change in pH plays a vital role in radical distribution and the efficiency of the process (Figure 4) [71]. The UV/persulfate process is more favourable under acidic condition due to enhanced $SO_{4}^{•\;-}$ generation. Under basic conditions the $SO_{4}^{•\;-}$ reacts with ^-^OH to generate ^•^OH, which is ineffective in PFAS degradation [72].

**Figure 5:**
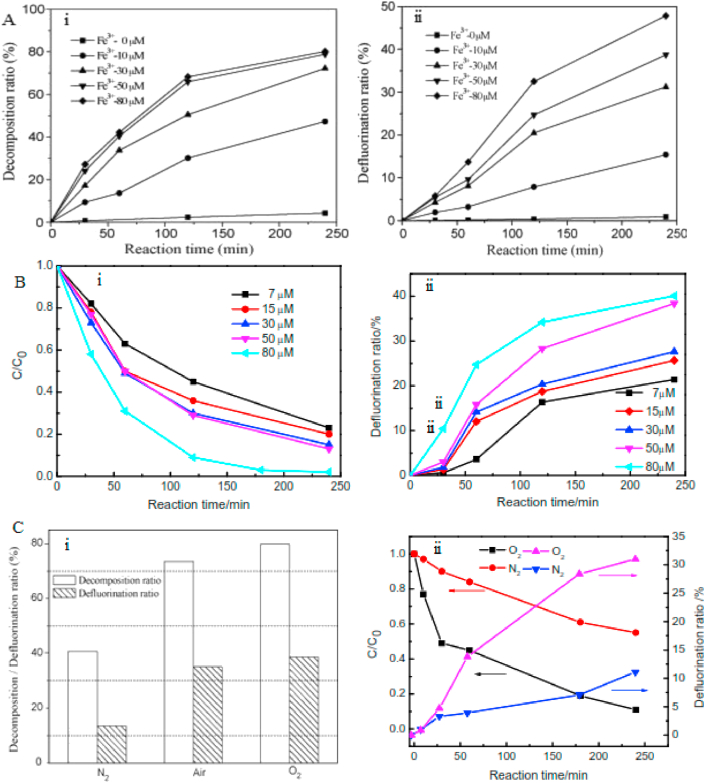
Effects of process parameters on the degradation and defluorination efficiency. (A) Effect of Fe^3+^ on degradation (i) and defluorination (ii) of PFOA in the presence of oxalic acid (B) Effect of initial Fe^3+^ on the degradation (i) and defluorination of PFOA (ii) (C) Effect of reaction atmosphere on the degradation and defluorination of PFOA in Fe^3+^(i) and Fe^3+^/oxalic acid (ii) Reproduced with permission from Wang *et al*. [74, 75]. Copyright (2008) Elsevier and Copyright (2016) Springer Nature.

Apart from persulfate, other oxidants like periodate, ferric ion, sodium hydrogen carbonate and coexisting ferric ion and oxalate have also been explored for PFAS photodegradation [73, 74, 75, 76]. The main drawbacks of the persulfate process are the large molar excess of the oxidant needed over the starting concentration of pollutant and the relatively long treatment time required by the process [12]. Most PFAS photochemical degradation process are influence by process parameters such as oxidant concentration, process atmosphere, and the presence of other ions in the system. Figure 5 shows the variation of photodegradation and defluorination efficiency of a ferric ion mediated decomposition of PFOA with process parameters.

### 3.3 Photocatalytic processes

In a photocatalytic system, three components are involved: the light source, catalyst and oxidant. The use of a catalyst makes this process admirable because it allows for the principles of catalysis to be explored in the degradation of contaminants. Photocatalysis is the most explored process for the degradation of PFAS, because the development of novel semiconductor catalytic materials has continued to be a major research focus for several decades.

One unique property of the photocatalytic process is that the catalyst undergoes a cyclic reaction and is, thus, regenerated at the end of the catalytic cycle. This allows for the reusability of the catalyst. The catalyst could either be in the same phase with the reaction system, in which case they are referred to as a homogeneous catalyst; or it could be in a different phase from the system, referred to as a heterogeneous catalyst. Homogeneous photocatalytic degradation of PFAS is based on the photo-Fenton process, which involves the generation of ^•^OH through the Fenton reaction between Fe^2+^ and H_2_O_2_ (Eqs. (3) and (4)) [77].

(3) Fe2++H2O2→Fe3++O•H+OH−

(4) $$Fe^{3+}+\;H_{2}O_{2}\;\to Fe^{2+}+\;HO_{2}^{•}+\;H^{+}$$

So far, the report by Tang *et al.* [78] on the degradation of PFOA via the photo-Fenton process, in which over 90% degradation and 53.2% defluorination was achieved after 5 h using an initial concentration of 20.0 μmol/L, seems the only significant study. The generation of ^•^OH and the reaction between PFOA and Fe^3+^ to form electron transfer complex were considered as the driving force behind the degradation process. Two degradation stages were identified: stage 1 involved the generation of reactive radical species mainly ^•^OH by the decomposition of H_2_O_2_ in the first 1 h of the reaction. This resulted in about 90% PFOA removal and a defluorination ratio of 35.8%. During the second stage of the process, degradation is driven by the activity of Fe^3+^ under UV irradiation which results in further degradation and increased defluorination to 53.2%. However, question have risen on the claim that the degradation was driven by the ^•^OH generated by the process, since there are many studies that have confirmed its unsuitability for PFAS degradation. The missing gap in this study is the inability of the authors to ascertain the PFOA excitation process in the photo-Fenton process. With recent studies showing the possibility of charge transfer between PFOA and Fe(III) to form a PFOA-Fe(III) complex, it is possible that this reaction occurred prior to the activity of ^•^OH in the reaction system [74, 75, 79].

For heterogeneous photocatalytic processes, a semiconductor material is activated by light radiation which is used in charge carrier generation. This then triggers a series of oxidation-reduction reactions. A wide range of semiconductors have been explored as photocatalysts for degradation of contaminants in wastewater, which include oxides (Bi_2_WO_3_, Ga_2_O_3_, SnO_2_, Ag_3_PO_4_, TiO_2_, and In_2_O_3_) [80, 81, 82, 83, 84, 85], sulphides (Ga_2_S_3_, CuS, and ZnS) [86, 87, 88] and nitrides (C_3_N_4_, and g-C_3_N_5_). Recently, semiconductor materials with band gaps capable of absorption in the visible range has allowed for the potential application of solar light for photocatalytic processes. Some of these semiconductors include: BiOBr [89], Ag/Bi_2_MoO_6_ [90] and Bi_2_WO_6_ [91]. The major semiconductors that have been extensively explored as photocatalysts for the degradation of PFAS are TiO_2_, In_2_O_3_ and Ga_2_O_3_ [92, 93, 94]. Other photocatalytic materials such as BiOCl and ZnO have also been explored for PFAS degradation [95, 96]. Photocatalytic degradation of PFAS is based on the generation of negatively charged electron (e^−^) and positively charged hole (h^+^) pairs, when light of appropriate energy (equal to or greater than the catalyst's band gap) is absorbed by the catalyst. These charge carriers then migrate to the surface of the catalyst to react with adsorbed molecules. Other reactive radical species such as ^•^OH and $O_{2}^{.\;-}$, are generated by the reaction of the electron-hole pair with adsorbed H_2_O and O_2_ (Eqs. (5), (6), (7), and (8); SP represents the semiconductor photocatalyst) [97]. Another merit of this systems is that dissolved oxygen present in the system serves as the oxidant, therefore no external oxidant is often required by the system.

(5) $$SP\;\overset{hv}{\to }\;SP\left(e^{-}+h^{+}\right)$$

(6) SPsurf(h+)+H2Oads→SPsurf+O•Hads+H+

(7) SPsurf(h+)+HO−→SPSurf+O•Hads

(8) $$SP\left(e^{-}\right)+\;O_{2}\;\to SP+\;O_{2}^{•-}$$

**Figure 6:**
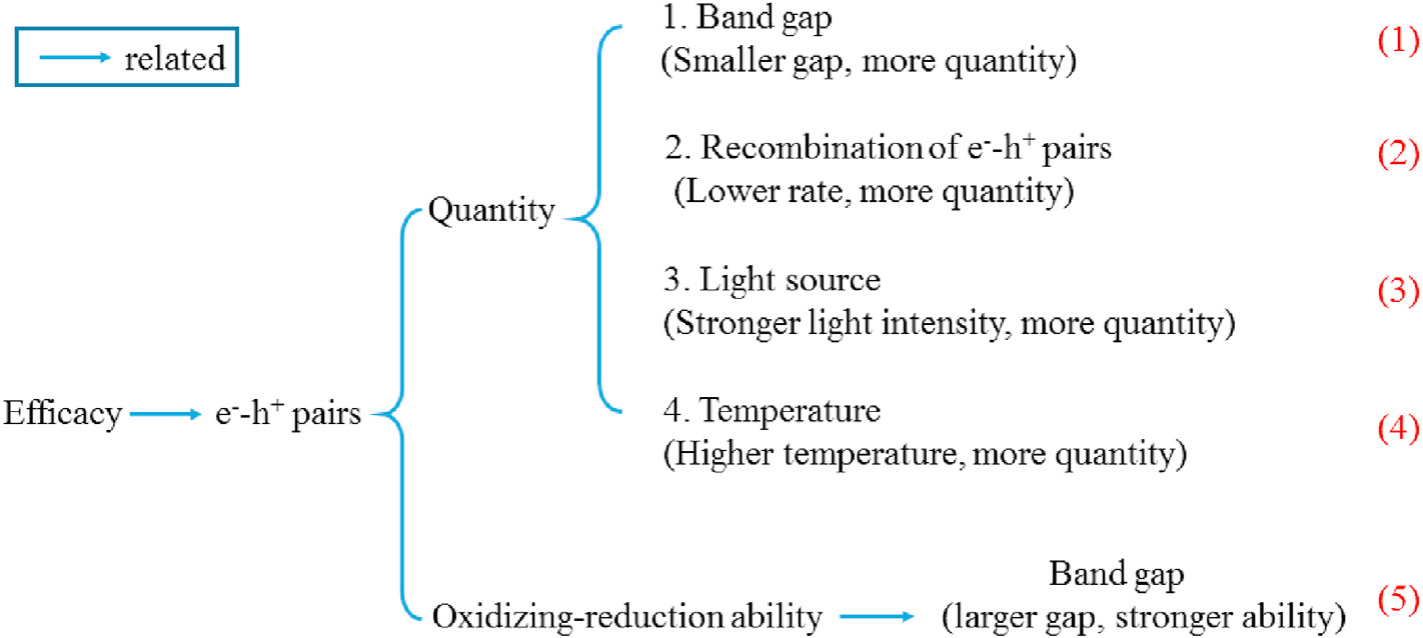
Correlation between process efficiency and the e^−^-h^+^ pair properties. Reproduced with permission from Xu *et al.* [98]. Copyright (2017) Elsevier.

However, for PFAS degradation, the reaction of h^+^ with dissolved oxygen is not desirable. This is because ^•^OH is not effective in PFAS degradation, while h^+^ is an effective oxidant for the degradation process. The nature of the catalyst's band gap is very important in the efficiency of the photocatalytic process as too narrow or too wide band gap will render the catalyst ineffective [98]. Figure 6 shows how the efficiency of the process correlates with the e^−^-h^+^ pair and the catalyst's properties. Photocatalysts with too narrow band gaps generate large quantities of e^−^-h^+^ pairs, but the oxidizing-reduction ability of the pair is weak. When the band gap is too wide, then only a few e^−^-h^+^ pair is generated since e^−^ transition from the valence band to the conduction band is minimized, even though the e^−^-h^+^ pair generated have high oxidation-reduction potential. This accounts for the continual quest for the development of catalysts with suitable band structure for PFAS degradation.

TiO_2_-based photocatalyst that have been explored for PFAS degradation have shown up to 100% degradation, depending on the properties of the catalyst such as band gap and surface area; and also process parameters such as light source, light intensity, catalyst dosage, pH and initial PFAS concentration [98]. Although, pristine TiO_2_ showed a very low degradation efficiency of 15% due to high e^−^-h^+^ pair recombination, its efficiency could be enhanced by the incorporation of hole scavengers like oxalic acid and perchlorate acid into the system [99, 100]. Degradation of 24 μM PFOA using TiO_2_ in the presence of oxalic acid, achieved 86.7% degradation and 16.5% defluorination under nitrogen environment after 180 min compared to 12.4% degradation and <1% degradation achieved in the absence of oxalic acid. Not only was the hole scavenging activity of oxalic acid significant in the reaction, but its generation of $CO_{2}^{•\;-}$ also influenced the degradation efficiency. The reaction rate constant was 11.6 times higher than what was obtained for the process carried out in the presence of HClO_4_ [99].

Modification of TiO_2_ with transition and noble metals (e.g Pd, Pt, Pb, Cu), carbon-based materials (e.g. carbon nanotubes) and polymers have also resulted in significant improvement in the degradation efficiency in the range of 45–100% [101, 102]. Chen *et al.* [101] reported the degradation of 50 mg/L of PFOA by Fe- and Cu- modified TiO_2_. The degradation efficiency of the Fe-TiO_2_ and Cu-TiO_2_ reached 69 and 91% respectively after 12 h under UV light. The defluorination efficiency also reached 9 and 19% efficiency for Fe-TiO_2_ and Cu-TiO_2_ respectively. This was a significant improvement in efficiency when compared to 14% degradation and negligible defluorination recorded for pristine TiO_2_. Although, no significant alteration in the band gap of TiO_2_ was observed in the doped materials, the enhanced activity was suggested to be due to reduction in the e^−^-h^+^ pair recombination process due to the possible trapping of the photogenerated electrons by vacancies in the doped catalysts. The rate constant for the degradation process was 0.0015 and 0.0031 min^−1^ for Fe- and Cu-TiO_2_ respectively. Surface modification of TiO_2_ catalyst induced by F^−^ was studied by Sansotera *et al.* [103] during the degradation of PFOA at concentration range above and below the critical micelle concentration (CMC). After 2 and 4 h of activity, a relatively constant fluorine concentration of 25.1 and 23.9% respectively was observed to be present on the catalyst surface. The interaction of fluorine with the catalyst resulted in morphological variation in TiO_2_, leading to reduced activity. This effect has been attributed to the stabilization of F^−^ around Ti^4+^, charge carrier mobility limitation and the formation of titanium hydroxyfluoride species [104].

In_2_O_3_-based photocatalysts are another class of materials that have been explored for PFAS degradation. Unlike TiO_2_, pristine In_2_O_3_ exhibits a high and fast degradation efficiency towards PFAS with degradation efficiency of 75–100% reported within 1 h by varying the morphology of In_2_O_3_ [105, 106, 107]. The outstanding catalytic performance of In_2_O_3_ have been attributed to the presence of high oxygen vacancy defects in its structure, which inhibits charge carrier recombination [108, 109]. The effect of morphology on the catalytic activity of In_2_O_3_ was studied by Li *et al.* [107]. This was carried out by evaluating the activities of In_2_O_3_ microspheres, nanoplates and nanocubes on the degradation of PFAS under UV light. Complete degradation of PFOA was achieved by the three In_2_O_3_ nanostructures after 20, 40 and 120 min for microsphere, nanoplates and nanocubes respectively. Evaluation of TiO_2_ activity in the same system only showed 28.5% degradation after 180 min. The rate constant for the degradation were 7.9, 4.5, 1.8 and 0.1 h^−1^ respectively for microspheres, nanoplates, nanocubes and TiO_2_ respectively. BiOCl nanosheet is another photocatalyst whose oxygen vacancies has been correlated with high defluorination efficiency which reached 59.3% after 3 h for degradation of 20 μmol/L of PFOA [95].

The photocatalytic activity of a material on the degradation of PFAS is influence by several environmental parameters such as the pH of the solution, catalyst dosage, presence of natural organic matter, reaction atmosphere and presence of inorganic ions in the system. Zhang *et al.* [110] confirmed that the degradation of PFAS is higher at low pH values and in the presence of low amount of sulphate and chloride. The degradation efficiency of a photocatalytic process is also influenced by the light source employed [111]. Evaluation of photodegradation efficiency of 50 mg/L by UV light (185 nm and 254 nm) and visible light (400–800 nm), showed that while complete degradation was achieved within 60 min under UV light radiation, the process required 120 min under visible light radiation. The activity of some reported advanced oxidation processes for PFAS degradation are shown in Table 3. The degradation efficiency for PFOA in the photooxidation processes ranged from 9-100%, while reported efficiency for PFOS is the range 12–95%. The defluorination efficiency range was 0.0–95.6% and 47.5–58.2% for PFOA and PFOS respectively. The addition of oxidants or semiconductor photocatalysts generally lead to an increase in the efficiency of degradation when compared to the direct photolytic processes.

**Table 3:** Advanced oxidation processes for the degradation of PFAS.

PFAS	Process	Light source	Process parameters	Degradation efficiency (%)	De-fluorination efficiency (%)	Kinetics	Process overview	Ref
PFOA	UV	Low-pressure Hg UV light (23W); Wavelength = 185 & 254 nm; intensity = 62–69 mW/cm^2^	|PFOA| = 0.004 mg/L;	9	-	1.5 × 10^−5^ h^−1^	The positive impact of $IO_{4}^{-}$ to the UV process was due to the generation of $IO_{3}^{•}$ which initiate the oxidation of PFOA, while under VUV, the $e_{aq}^{-}$ scavenges the $IO_{4}^{-}$, thus reducing reactive radical generation.	[73]
	UV/NaIO_4_			70	17	1.6 × 10^−4^ h^−1^		
	VUV		|NaIO_4_| = 0.5 mM Time = 120 min	87	25	3.0 × 10^−4^ h^−1^		
	VUV/NaIO_4_			60	13	2.0 × 10^−4^ h^−1^		
PFOA	UV/TiO_2_	400W UV lamp; Wavelength = 254 nm	|PFOA| = 50 mg/L; |Catalyst| = 0.5 g/L	18.3	0.0	1.5 × 10^−3^ h^−1^	Pb-doped TiO_2_ exhibited high degradation and defluorination efficiency due to the production of traps to capture photo-induced e^−^ or h^+^	[92]
	UV/Pb-TiO_2_		Time = 12 h; pH = 5	99.9	22.4	5.1 × 10^−1^ h^−1^		
PFOA	VUV	Low-pressure Hg UV light (12W); Wavelength = 185 & 254 nm	|PFOA| = 0.01 mg/L; |Fe^3+^| = 36 μM	80	20.0	2.0 × 10^−5^ h^−1^	The presence of Fe^3+^ catalyzed the activation of PFOA under VUV radiation, leading to improved defluorination efficiency	[112]
	VUV/Fe^3+^		Time = 4 h; pH = 5		51.2	5.0 × 10^−5^ h^−1^		
PFOA	UV	medium-pressure Hg UV light (150W); Wavelength = 200–600 nm	|PFOA| = 0.1 mg/L; |Catalyst | = 0.1 g/L	24	-	-	The photodegradation activity of TiO_2_ was enhanced by incorporating rGO, which reduces electron/hole recombination by capturing photogenerated electrons	[113]
	UV/TiO_2_			58	-	-		
	UV/TiO_2_-rGO		Time = 12 h; pH = 3.8	93	20	0.16 h^−1^		
PFOS	UV	Low-pressure Hg UV light (23 W); Wavelength = 254 nm	|PFOS| = 0.01 mg/L; |Fe^3+^| = 100 μM	12	-	1.3 × 10^−3^ h^−1^	The addition of Fe^3+^ increased the reaction rate by almost 50 times. This was due to the formation of a Fe^3+^-PFOS complex, which is excited and photolyzed by UV light	[114]
	UV/Fe^3+^		Time = 48 h; pH = 3.6	98	58.2	7.0 × 10^−2^ h^−1^		
PFOA	UV/$\;NO_{3}^{-}$	Low-pressure Hg UV light (18 W); Wavelength = 254 nm	|PFOA| = 5 mg/L; |$\;NO_{3}^{-}$| = 100 mM	23.7	-	0.03 h^−1^	Irradiation of UV light with 254 nm yields peroxynitrous acid from nitrate, which decomposes reversibly to form $NO_{2}^{•}$ and O•H. The isopropanol scavenges the O•H, resulting in increased $NO_{2}^{•}$ formation	[115]
			|Pr-OH| = 0.012 ppm					
	UV/$NO_{3}^{-}$/Propanol		Time = 11 h; pH = 6.3	100	95.6	0.27 h^−1^		
PFOA	UV/TiO_2_	high-pressure Hg UV light (125 W); Wavelength = 254 nm; intensity = 5.3 mW/cm^2^	|PFOA| = 60 mg/L |M-TiO_2_| = 0.5 g/L	31.1	3.3	0.06 h^−1^	The noble metal doped TiO_2_ exhibited improved efficiency by acting as electron sinks. Metals with larger work function (Pt and Pd) showed higher activity because of their efficiency in capturing of electrons	[102]
	UV/Ag-TiO_2_			57.7	8.1	0.13 h^−1^		
	UV/Pd-TiO_2_		Time = 7 h; pH = 3.0	94.2	25.9	0.44 h^−1^		
	UV/Pt-TiO_2_			100	34.8	0.73 h^−1^		
PFOA	UV/BiOI	high-pressure Hg UV light (300W); Wavelength = 400–600 nm	|PFOA| = 20 mg/L |Cat| = 12 mg/L	66	-	8.0 × 10^−5^ h^−1^	The enlarged surface area of the Br-doped BiOI, resulted in enhanced UV-light absorption and increased electron-hole separation.	[33]
	UV/BiOBr		Time = 2 h; pH = 3.0	96	-a	3.3 × 10^−4^ h^−1^		
PFOA	UV/In_2_O_3_	Low-pressure Hg UV light (15 W); Wavelength = 254 nm	|PFOA| = 30 mg/L |Cat| = 0.05 g/L	87	29.7	-	The study on the effect of compositing In_2_O_3_ with graphene and the effect of calcination temperature on the activity of the catalyst showed that improved activity was obtained when the catalyst was calcined at 400 °C	[116]
	UV/In_2_O_3_-G100			29	12.9	-		
	UV/In_2_O_3_-G350		Time = 3 h;	87	37.7	-		
	UV/In_2_O_3_-G400			100	60.9	-		
PFOA	UV	high-pressure Hg UV light (400 W); Wavelength = 254 nm; intensity = 5.3 mW/cm^2^	|PFOA| = 50 mg/L |Cat| = 40 mM	52.1	38.3	-	Under UV irradiation $CO_{3}^{•\;-}$ by the reaction of ^•^OH with $HCO_{3}^{-}$. The $CO_{3}^{•\;-}$ oxidize the perfluorinated carboxyl anions to form perfluorinated alkyl radicals.	[76]
			Time = 12 h					
	UV/NaHCO_3_		pH = 8.8	100	82.3	0.37 h^−1^		
PFOA	UV-A/Fe-zeolites	Hg UV-A light (4 W); Wavelength = 254 nm; Photon flux = 4.47 × 10^−6^ mol/s^−1^	|PFOA| = 0.02 mg/L, |Cat| = 1 g/L Time = 24 h	100	38	0.38 h^−1^	PFOA is adsorbed by the catalyst and forms PFOA-Fe^3+^ complex. Under irradiation the PFOA is oxidized through a ligand-to-metal charge transfer process	[117]
			pH = 5.5					
PFOA	UV/β-Ga_2_O_3_	Low-pressure Hg UV light (18 W); Wavelength = 254 nm	|PFOA| = 0.05 mg/L |Cat| = 1.8 g/L Time = 2 h	70	30	0.44 h^−1^	A novel catalyst was developed with high activity for PFOA degradation. Activity was related primarily to the surface charge of the material and its charge carriers favorable redox potentials. Catalytic activity was not inhibited at low concentration nor in the presence of NOMs	[118]
	UV/BiPO_4_	Light intensity = 2.17 × 10^−5^ Es.L^−1^,S^−1^	pH = 5.5	72	39	0.40 h^−1^		
	UV/BOHP			100	63	6.0 h^−1^		
PFOA	UV/Pb-BiFeO-rGO	Low-pressure Hg UV light (5 W); Wavelength = 254 nm	|PFOA| = 50 mg/L |Cat| = 100 mg/L Time = 8 h	70	37.6	0.08 h^−1^	The effect of the amount of rGO on the photocatalytic activity showed that optimum amount of rGO was 0.5 w%, which gave the highest activity for PFOA degradation	[119]
			pH = 2.0					
PFOA	Vis/FeO-CS	Solar simulator; Light intensity = 100 mW/cm^2^	|PFOA| = 0.3 mg/L |Cat| = 1.0 g/L Time = 4 h	95.2	57.2	-	The prepared catalyst showed high adsorption properties, which facilitated the degradation of PFOA through direct electron extraction under light irradiation from PFOA	[120]
			pH = 7.0					
1H, 1H, 2H, 2H PFOS(6:2FTS)	UV/Fe^3+^	Low-pressure mercury lamp (23 W) Wavelength = 254 nm	|PFOS| = 10 mg/L, |Fe^3+^ | = 100 μmol/L,	95	48.4	1.56 h^−1^	Degradation of PFOS was achieved by either the attack of ^⋅^OH on 6:2FTS, resulting in C-C bond cleavage or the cleavage of C-S bond in the Fe(III)-6:2FTS complexes through ligand-to-metal charge transfer when irradiated with light	[121]
			Time 3 h					
			pH = 3.0,					
PFOS	UV/Fe^3+^	Low pressure mercury lamps (23 W), Wavelength = 254 nm (UV)	|PFOS| = 0.01 mg/L	85.3	47.5	3.2 × 10^−2^ h^−1^	While the degradation efficiency of the UV process was greatly inhibited by the presence of organic matter, the VUV showed no significant change in efficiency in the presence of organic matter	[122]
			|Fe^3+^| = 100 μM					
			Time = 24 h					
			pH = 3.0					

a HP = H_3_PW_12_O_40.6_H_2_O, BOHP = Bi_3_O(OH)(PO_4_)_2_; CS = carbon sphere.

## 4 Advanced reduction processes for PFAS degradation

Advanced reduction process (ARP) is a new class of degradation process that combines activation methods (UV light, electron beam, microwave and ultrasound) and reducing agents (sulphite, sulphide, ferrous iron and dithionite) to generate highly reactive reducing radicals [123]. The main reducing radicals generated are $e_{aq}^{-}$ and H^•^ alongside other radicals such as $SO_{3}^{•\;-}$ and $SO_{2}^{•\;-},$ depending on the activation methods and the type of solutes present in the system [55]. The efficiency of this process is mostly ascribed to the hydrated electron, which is a strong reducing agent, with strong negative reduction potential of -2.9 V. Hydrated electrons have proven to be a fast and effective reducing agent for PFAS degradation. The most explored activation/reducing agent combination for PFAS degradation are the UV/KI and UV/$SO_{3}^{•\;-}$ system.

In the UV/sulphite system, $e_{aq}^{-}$ , $SO_{3}^{•\;-}$ and H^•^ are the generated radicals as shown in Eqs. (9) and (10). The $SO_{3}^{•\;-}$ generated is converted to $\;S_{2}O_{6}^{2-}$, $SO_{4}^{2-}$ and $H^{+}$ (Eqs. (11) and (12)), so it makes no significant contribution to the system [124, 125].

(9) $$SO_{3}^{2-}+hv\;\to \;e_{aq}^{-}+\;SO_{3}^{•\;-}$$

(10) $$HSO_{3}^{-}+hv\;\to H^{•}+\;SO_{3}^{•-\;}$$

(11) $$SO_{3}^{•\;-}+\;SO_{3}^{•\;-}\;\to S_{2}O_{6}^{2-}$$

(12) $$SO_{3}^{•\;-}+\;SO_{3}^{•\;-}+\;H_{2}O\;\to SO_{3}^{2-}+\;SO_{4}^{2-}+2H^{+}$$

Gu *et al.* [126] reported the use of a high photon flux (9.93 × 10^−8^ E/cm^2^.s) with sulphite in the decomposition of 32 μM PFOS. A high degradation efficiency of 98% and reaction rate of 0.118 min^−1^ were achieved within 30 min. The rapid and high degradation was due to high $e_{aq}^{-}\;$ generation induced by the high photon flux. In addition, the process tolerated high concentration of nitrate and other inorganic solutes with scavenging effect on $e_{aq}^{-}.$ The importance of the photon flux in the system was further confirmed by reducing the intensity of the incidence light by 70%. This resulted in a significant reduction in the reaction rate constant to 0.020 min^−1^. In another study conducted under VUV light and with sulphite ions, about 97.3% degradation of 37.2 μM PFOS was achieved within 4 h, and a high defluorination efficiency of 75.4% was recorded. The reaction rate constant was 0.87 h^−1^, which was about 8 and 2-folds the reaction rate for VUV and UV/sulphite processes. With VUV/sulphite process, a high degradation efficiency was still achieved even at both weak acidic and alkaline pH [127].

**Figure 7:**
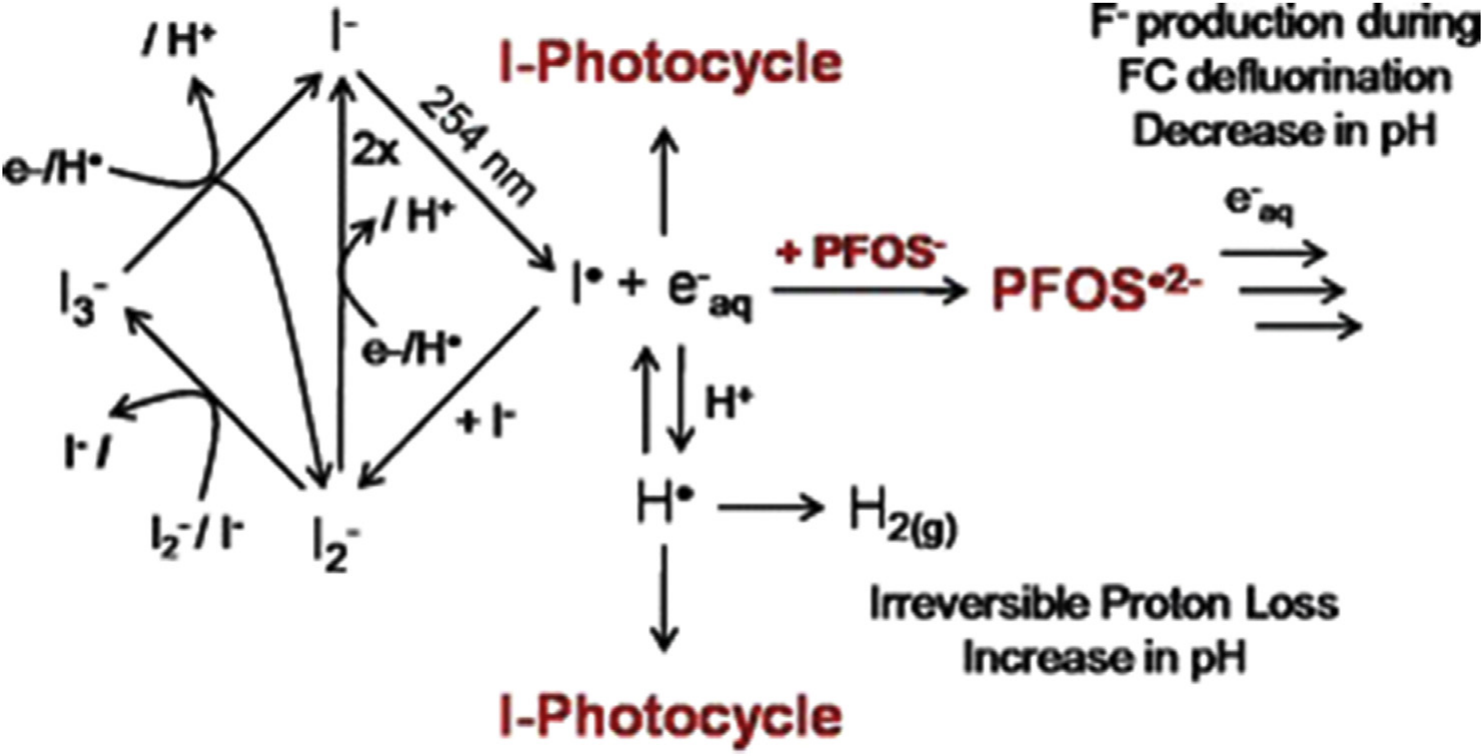
Iodide photocycle in the UV/KI process. Reproduced with permission from Park *et al.* [128]. Copyright (2011) Royal Society of Chemistry.

The UV/I^−^ is another ARP process that has been explored in the degradation of PFAS. The $e_{aq}^{-}$ generation occurs via “charge-transfer-to-solvent states”, with a quantum yield of 0.286 on a femtosecond scale. Figure 7, shows a schematic representation of possible reaction routes to the formation $e_{aq}^{-}$ in the UV/KI system.

Qu *et al.* [129] studied the effect of the initial pH of the system on the degradation efficiency of UV/I^−^ on PFOA. The report showed that the process degradation and defluorination efficiencies depended strongly on the solution pH. Evaluating the system within the pH range of 5–10, it was observed that the reaction rate at pH 10, was 49 times more than the rate observed at pH 5. The toxicity of the generated intermediates was also reduced at high pH, because lower concentration of short-chain PFCAs was reached within a short reaction time.

Reductive degradation of six perfluoroalkyl compounds including perfluorooctanoate (PFOA), perfluorohexanoate (PFHA), perfluorobutanoate (PFBA), perfluorooctane sulfonate (PFOS), perfluorohexane sulfonate (PFHS) and perfluorobutane sulfonate (PFBS) was studied using the UV/KI [128]. Their degradation was found to be dependent on the perfluorocarbon chain length, iodide concentration, headgroup and the initial concentration. The process was also independent on the pH as no significant change in kinetics was observed between the pH 2–12 in contrast to other reported studies [128, 129]. In a similar study, Park *et al.* [130] evaluated the effect of ionic headgroup and chain length on three perfluoroalkyl carboxylates (PFBA, PFHA and PFOA) and three PFSAs (PFHS, PFBS and PFOS) by the UV/KI process. The study showed that, for the perfluoroalkysulfonate with -SO_3_^-^ headgroup, the reduction kinetics and extent of defluorination increased with increase in chain length, while the chain length had no significant influence on both kinetics and extent of reduction for the PFCAs. The extent of reactions, which was measured as the fraction of flourine ion produced to initial PFC, were 1.2, 1.9, 1.6, 2.5, 5.9, and 9.2 for PFBA, PFHA, PFOA, PFBS, PFHS and PFOS respectively, showing that the PFCs with sulfonate headgroups were easily degraded compared to the ones with the carboxylate head groups.

**Figure 8:**
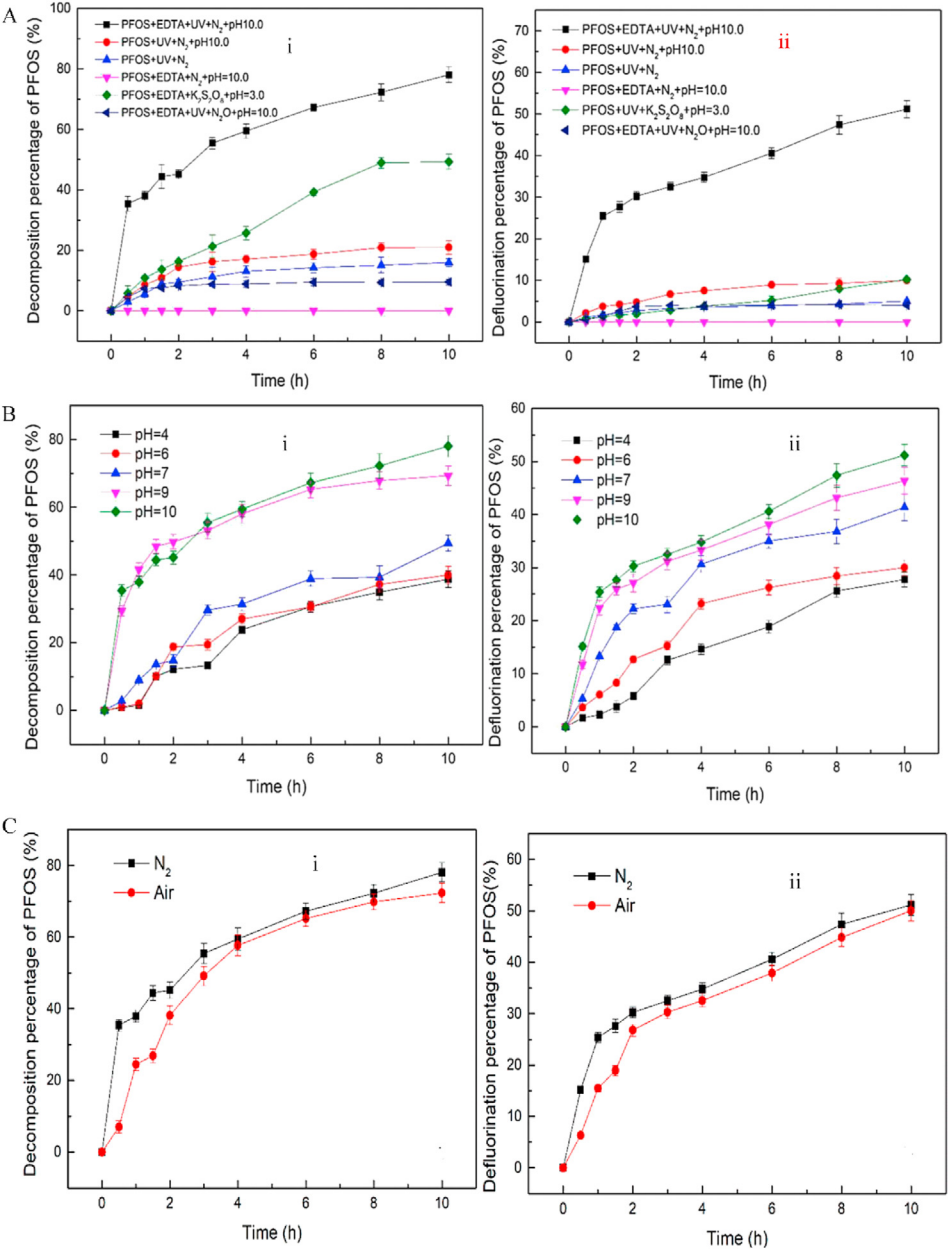
Effect of process parameters on the degradation and defluorination efficiency of PFOS by UV/EDTA (A) Variation of degradation (i) and defluorination (ii) efficiency with time under different conditions (B) Effect of pH on degradation (i) and defluorination (ii) efficiency (C) Effect of air on degradation (i) and defluorination (ii) efficiencies (d) reaction kinetics of UV/EDTA process and Toxicity study (ii) of degradation products. Reproduced with permission from Gu *et al.* [132]. Copyright (2020) Elsevier.

Recently, in order to circumvent the potential generation of undesirable by-products such as polyiodide, iodate (from the UV/I^−^ process) [131] and the introduction of sulphite, which is known for its toxicity on the reproductive and peripheral organs [132], attempts have been made to explore catalyst-free processes or the use of hole scavenging species in advanced reduction process. For example, Lyu *et al.* [53] reported that increased amount of $e_{aq}^{-}$ could be generated via photolysis by increasing the pH and temperature of the system, and by adding ROS scavengers such as t-BuOH. The catalyst free process achieved a degradation efficiency higher than 70% and a high reaction rate constant of 2.78 h^−1^ and 0.058 h^−1^ was attained at pH 7.0 and 90 °C for PFOA and PFOS degradation respectively. Figure 8, shows the correlation plots between the efficiency (degradation and defluorination) and process parameters for UV/ethylenediaminetetraacetic acid process (EDTA), with the EDTA acting as ROS scavengers. The study showed that direct photolysis only achieved 15.97 and 4.99% degradation and defluorination efficiency, which was increased to 20.97 and 9.99% when the pH of the system was adjusted to 10. The introduction of EDTA into the system at pH 10 resulted into improved degradation and defluorination efficiencies of 78.08 and 51.19% respectively. The removal of oxidizing species from the system resulted into a significant increase in the efficiency of the reductive process. Both the degradation and defluorination efficiencies were strongly influenced by the pH of the system, while the influence of the systems atmosphere showed no substantial influence on the process as shown in Figure 8b and c. The degradation of some PFAS by the ARP are presented in Table 4. The table shows that degradation efficiency ranging from 40-100% was achieved for PFOA, while PFOS degradation efficiency ranged between 46.2 and 97.9%. The defluorination efficiency was in the range 5–80.9% for PFOA, and 30–100% was recorded for PFOS. The degradation efficiency of PFAS is also significantly influenced by the nature of light source employed in the study. From the studies, UV light proved to be the most effective light source for photoreductive degradation of PFAS.

**Table 4:** Advanced reduction processes for the degradation of perfluoro alkyl substances.

Pollutant	Process	Light source	Process parameters	Degradation efficiency	Defluorination efficiency	Kinetics	Process overview	Ref.
PFOA	UV/KI	Low-pressure Hg UV light (15 W; Wavelength = 254 nm; Photon flux = 9.9 × 10^−6^ mol L^−1^ min^−1^)	|PFOA| = 0.008 mg/L; |KI| = 0.60 mmol/L; Temperature = 293–313 K; Ionic strength = 2.5–20 mmol/L; pH = 9; Time = 6 h	100	47.7–80.9%	3.2 × 10^−1^ – 1.5 h^−1^	Effect of temperature and ionic strength on the degradation of PFOA was studied. The efficiency showed positive correlation with both temperature and ionic strength	[133]
PFOS	VUV	VUV:Low pressure Hg lamp (10 W; wavelength = 254 nm and 185 nm)	|PFOS| = 0.2 mg/L; |$SO_{3}^{•\;-}$| = 0.60 mmol/L; pH = 10; Time = 4 h	46.2%	30%	1.2 × 10^−1^ h^−1^	The effect of light source in UV/sulphite and VUV/sulphite process showed the VUV/sulphite showed higher degradation efficiency due to the increased amount of generated reactive species	[134]
	UV/$SO_{3}^{•\;-}$			85.8%	64.6%	4.8 × 10^−1^ h^−1^		
	VUV/$SO_{3}^{•\;-}$	UV: low pressure Hg lamp (11W; wavelength: 254 nm)		97.3%	68.5%	8.7 × 10^−1^ h^−1^		
PFOS	UV/NTA	Low-pressure Hg UV light (14 W; Wavelength = 254 nm; Photon flux = 9.9 × 10^−6^ mol L^−1^ min^−1^)	|PFOS| = 0.005 mg/L; |NTA| = 2 mM; pH = 10; Time = 10 h	85.4%	46.8	2.7 × 10^−1^ h^−1^	The UV/NTA process showed high efficiency as NTA not only act as photosensitizer to induce water photodissociation and photoionization but act as ^•^OH scavenger, thus reducing its recombination with hydrated electron	[135]
PFOA	Vis/IAA/HDTMA-Montmorillonite	Low-pressure Hg UV light (36 W; Wavelength = 254 nm; Photon flux = 4.5 mW cm^−2^)	|PFOA| = 10 mg/L; |IAA| = 1 mM; |clay mineral| = 2.2 g/L pH = 3; Time = 5 h	100%	90% (within 10 h of reaction)	-	The formation of hydrated electrons was promoted by the stabilizing effect of the montmorillonite clay on the indole radical cation generated by photolysis. The hydrated electrons interact with adsorbed PFOA molecules in the interlayer of the clay.	[136]
PFOS	UV/KI UV/KI/HA	Low-pressure Hg UV light (14 W; Wavelength = 254 nm; Photon flux = 4.5 mW cm^−2^)	|PFOS| = 0.02 mg/L; |KI| = 0.3 mM; |HA| = 1 mg/L pH = 10; Time = 2 h	73.9%	44.4%	0.96 h^−1^	Enhanced degradation of PFOS was achieved in the presence of humic acid due to the increased generation of hydrated electrons; improved electron transfer due to certain HA functionalities; absorption of UV photons by HA to produce hydrated electrons	[137]
				97.9%	77.0%	1.1 h^−1^		
PFOS	UV/EDTA	Low-pressure Hg UV light (14 W; Wavelength = 254 nm; Photon flux = 4.5 mW cm^−2^)	|PFOS| = 0.005 mg/L; |EDTA| = 2.0 mM; |HA| = 1 mg/L pH = 10; Time = 10 h	78.08%	51.19%	1.13 × 10^−1^ h^−1^	EDTA acted primarily as OH radical scavenger. This scavenging activity allowed for the degradation of PFOS over a wide range of pH and in the presence of oxygen. Higher degradation and defluorination efficiency were also achieved for long chain PFOS.	[132]
Linear PFOS	VUV	Low-pressure Hg UV light (23 W; Wavelength = 185 nm; Photon flux = 1.8 mW cm^−2^)	|PFOS| = 10 mg/L; pH = 12.5; Time = 3 h (72 h was used for linear PFOS)	70	100	1.7 × 10^−2^ h^−1^	Reductive degradation of linear and branch was carried out under anoxic conditions. The degradation efficiencies showed that branched PFOS are easily degraded than linear PFOS. The active radical in the system was confirmed to be hydrated electrons	[138]
Branch-PFOS				91	96.2	1.3 × 10^−3^ h^−1^		
PFOA	UVC/KI	Low-pressure Hg UV light (20 W for UVC and 110 W for VUV); Wavelength = 185 nm (UVC) and 185 + 254 nm (VUV); Photon flux = 1.8 mW cm^−2^	|PFOA| = 10 mg/L; |KI| = 0.3 mmol/L pH = 5.5; Time = 3 h	40	~5	22.2 × 10^−2^ h^−1^	The effect of light source on the photoreductive degradation of PFOA was evaluated. The low efficiency of the UVC/KI system was attributed to the photons of the system being solely used for KI reduction, which eliminates the possibility of direct photolysis of PFOA	[139]
	VUV/KI			82	~10	50.4 × 10^−2^ h^−1^		

## 5 Degradation products and pathways

### 5.1 Advanced oxidation processes

In order to understand the photodegradation process of PFAS and improve on the process, it is important to evaluate the by-products obtained in the system and also explore the pathway to these products. Monitoring of by-products are also important in order to evaluate the change in toxicity achieved by the process.

The oxidative degradation of PFAS has been reported to follow a pattern involving, firstly electron transfer, which could be initiated by $SO_{4}^{•-},\;$ h^+^ or ^•^OH in photo-enhanced processes to give either radical species (PFASs^•^) or anionic radical (PFASs^• –^) [68, 78, 100]. This is followed by C-CO_2_H bond cleavage in PFCAs (decarboxylation) or C-SO_3_H bond cleavage in PFSAs (desulfonation) yielding unstable perfluoroalkyl radicals (C_n_F_2n+1_)^•^ [65, 140]. In photolytic processes, the direct cleavage of the C-CO_2_H and C-SO_3_H is achieved by the absorption of light usually in the VUV range [51]. The unstable perfluoroalkyl radical can either react with water to form a thermally unstable alcohol C_n_F_2n+1_OH [141, 142] or react with molecular oxygen generated in the system to yield perfluoroperoxy radical, which then undergoes a two-stage combination reaction with another perfluoroperoxy radical to yield the perfluorinated alcohol [143]. The choice of the route to the formation of the unstable alcohol species is reportedly dependent on the type of system employed and the pH of the reaction system [144, 145, 146]. The obtained alcohol then undergoes hydrogen fluoride elimination to form C_n-1_F_2n-1_COF, followed by CF_2_ elimination via hydrolysis to yield a shorter chain PFAS (C_n-1_F_2n-1_COOH). This short chain PFAS then undergoes, repeatedly, the chain reactions leading to CF_2_ elimination till complete mineralization is achieved [147]. This mechanism is referred to as the decarboxylation-hydroxylation-elimination-hydrolysis (DHEH) pathway and it is the most probable pathway for explaining the degradation and defluorination of PFAS.

**Figure 9:**
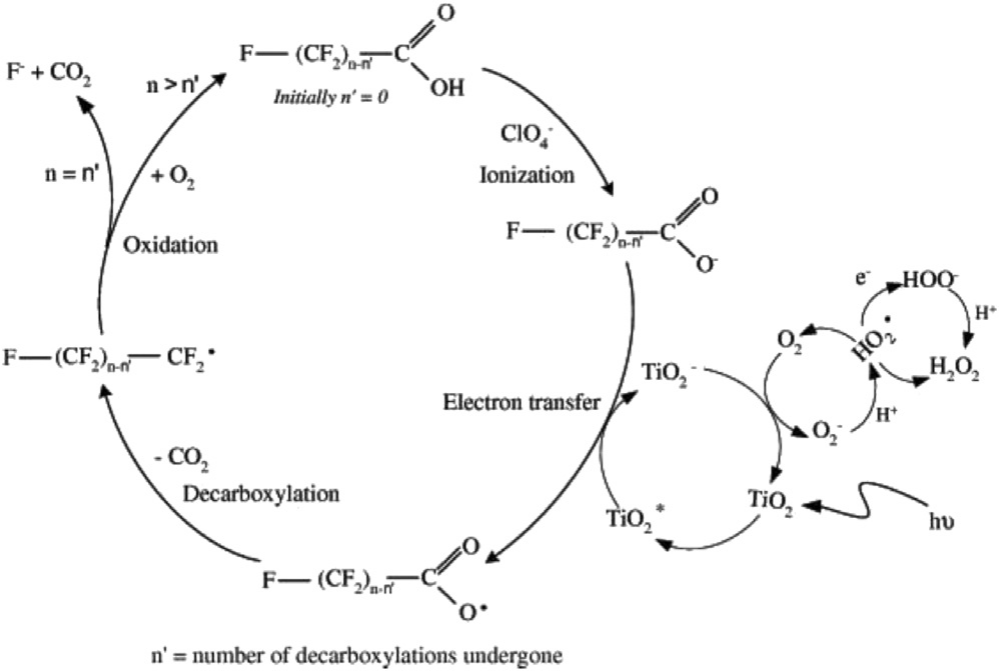
Photodegradation of PFOA by TiO_2_ photocatalysis. Reprinted with permission from Panchangan *et al*.) [100]. Copyright (2009) Elsevier.

The decomposition mechanism of PFCAs by heterogeneous photocatalysis reported by Panchangam *et al.* [100] is shown in Figure 9. The formation of the unstable alcohol specie was proposed to be through the reaction of perfluoroalkyl radical with water due to the prevailing acidic condition of the reaction system. The cycle of ionization, electron transfer, decarboxylation and oxidation of perfluoroalkyl radical continues, until the number of decarboxylation cycles (n′) becomes equal to the number of –(CF_2_)- chain group (n) in the target pollutant. That is, complete mineralization is achieved, otherwise short chain perfluoroalkyl compounds are formed in the reaction system. Some of the short chain PFCAs identified in the degradation of perfluorodecanoic acid (PFDA) in the study include perfluorobutanoic acid (PFBA), perfluoroheptanoic acid (PFHpA), perfluoropentanoic acid (PFPeA) and perfluoropanoic acid (PFPA).

**Figure 10:**
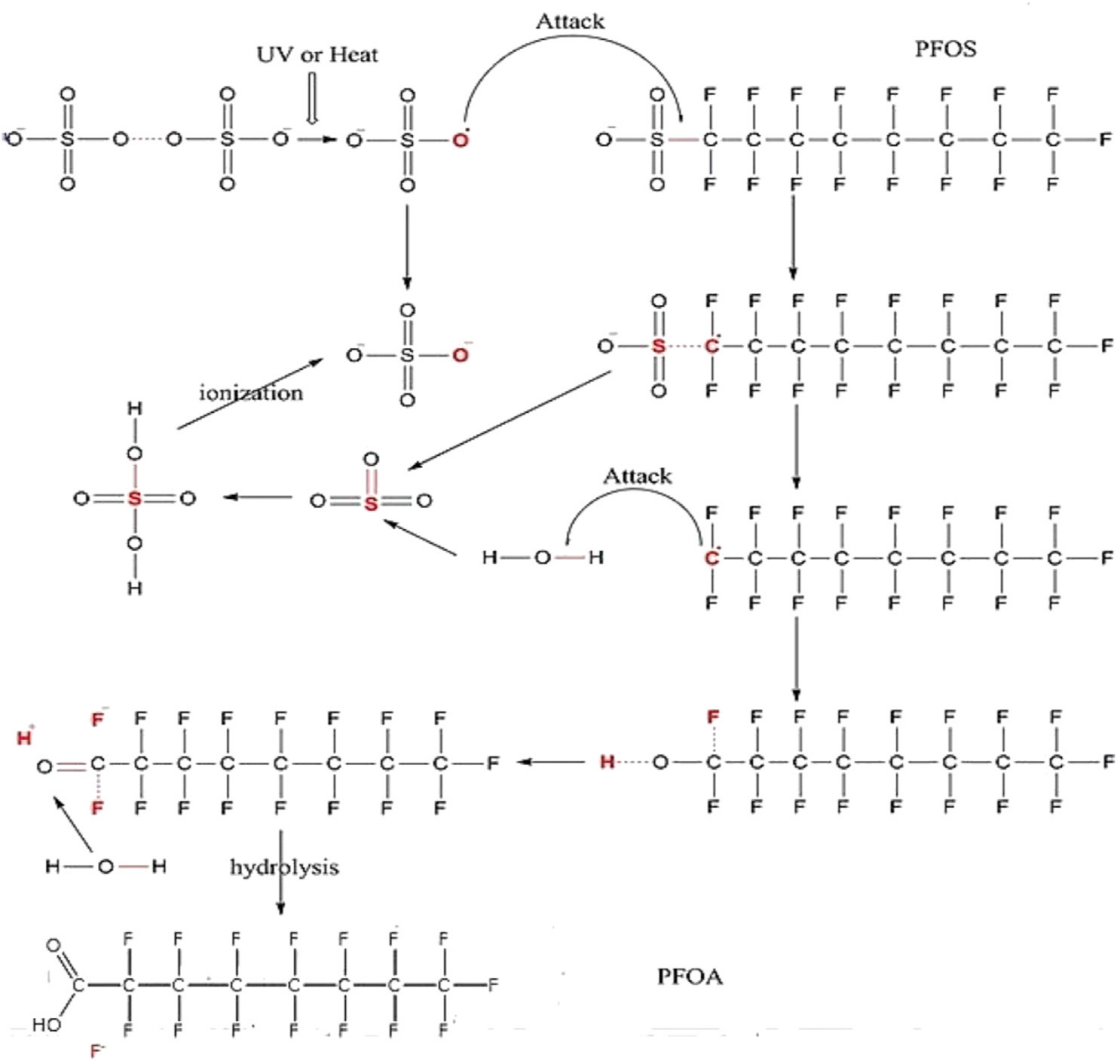
Photodegradation mechanism of PFOA degradation by UV/S_2_O_8_^-^ process. Reproduced from Yang *et al.* [68] under the creative common attribution licence.

For the defluorination process, which is very significant in achieving by-products with lower toxicity than the parent molecule, different pathways including, H/F exchange, hydrolysis, HF elimination triggering decarboxylation and dissociation of terminal functional group have been reported [148]. Yang *et al.* [68] reported in their study of the defluorination mechanism of PFOS in the UV/sulphite process, that the initial attack of $SO_{4}^{•-}$ on the PFOS molecule was the oxidation of the C-S bond, since the C-F cannot be easily oxidized by the radical (Figure 10). The unstable $C_{8}F_{17}^{•}$ formed reacts with $H_{2}O$, producing $C_{8}F_{17}OH$, which then undergoes HF elimination to produce $C_{8}F_{15}COF$. Short chain perfluorocarboxylic acids such as PFHpA, PFHxA, PEPA and PFBA were produced by the hydrolysis reaction between $C_{8}F_{17}COF$ and water, which forms short chain PFOA.

**Figure 11:**
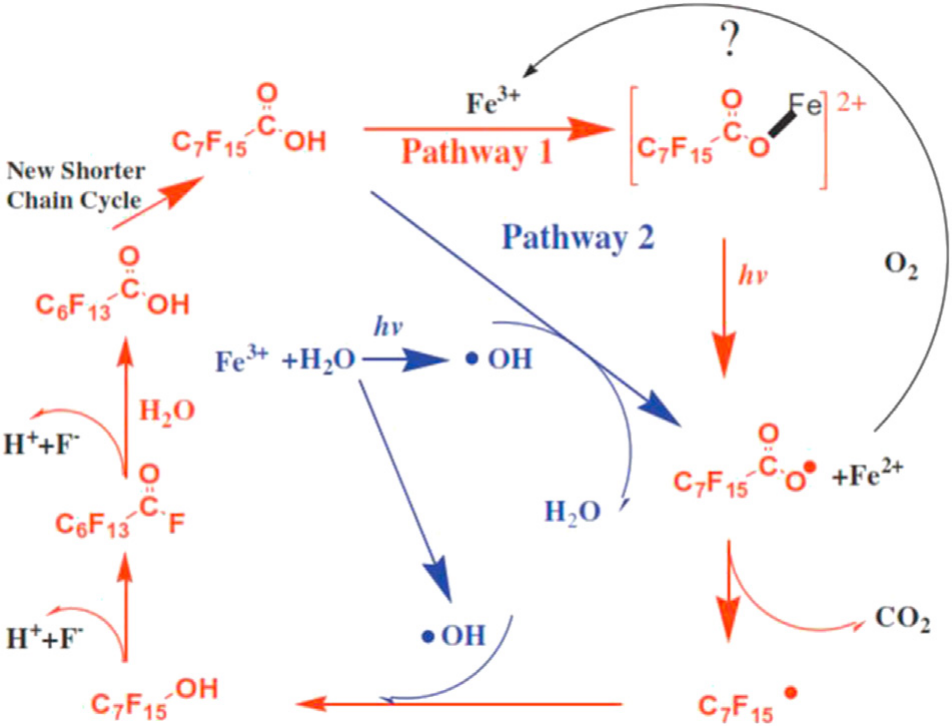
Reaction pathway for Iron-mediated degradation of PFAS under UV light radiation. Reproduced with permission from Liu and Xiu [79]. Copyright (2013) Elsevier.

For iron based photochemical processes, two simultaneous initiation pathways were proposed by Liu *et al.* [79] for the degradation of PFOA. In the first pathway, when irradiated with UV light, electron transfer occurs between PFOA and Fe(III) to yield an unstable radical $C_{7}F_{15}COO^{•}$ and Fe(II) via a PFOA-Fe(III) complex intermediate, which undergoes photolysis to give PFAS^•^ and Fe^2+^ on absorbing light energy. Simultaneously, under UV light radiation, Fe(III) reacts with H_2_O to form ^•^OH, which oxidizes PFOA to also form $C_{7}F_{15}COO^{•}$. The unstable radical then undergoes the DHEH pathway to give shorter chain PFOA as shown in Figure 11.

**Figure 12:**
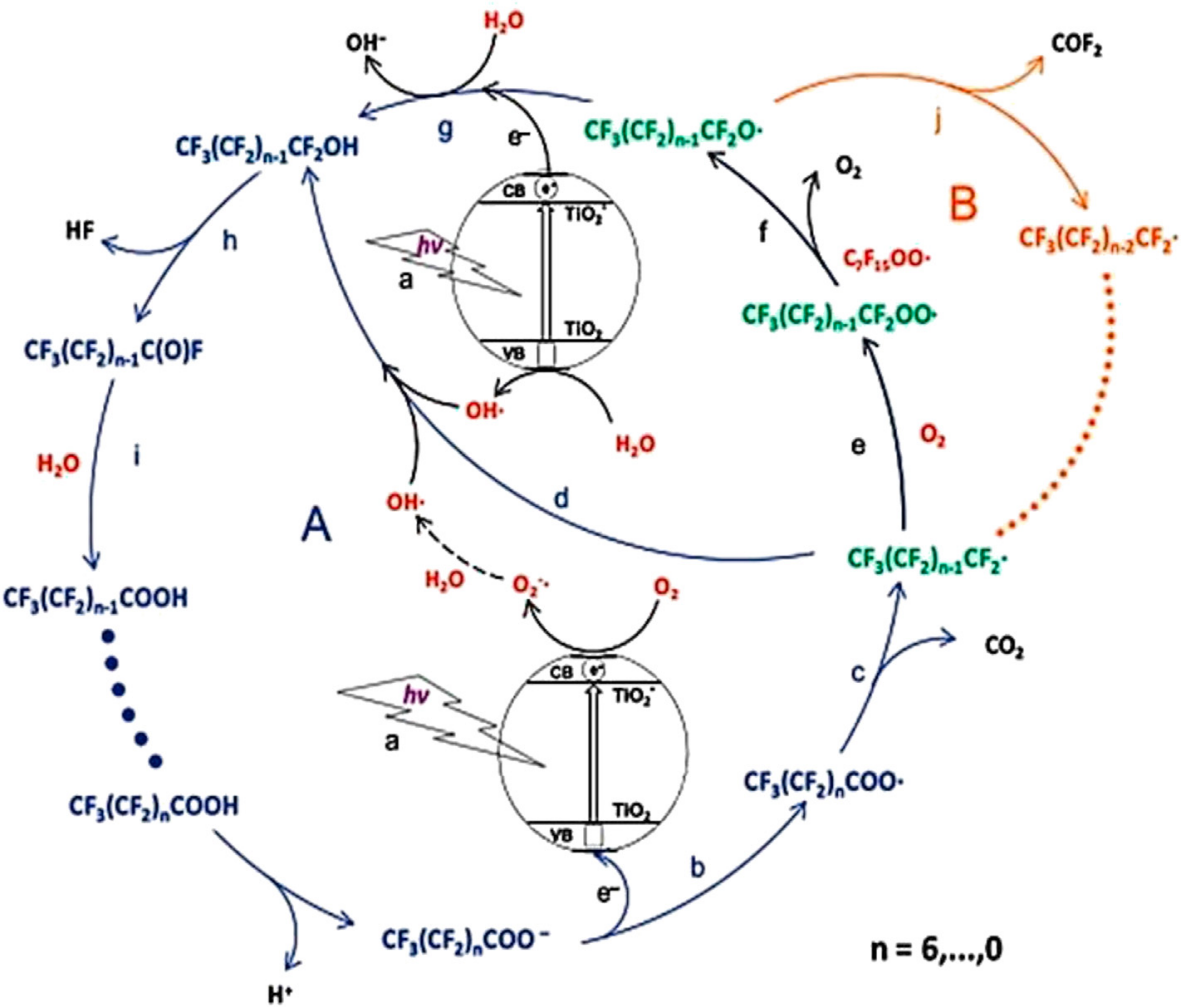
Effect of oxygen on the degradation pathway of PFOA. Green reaction intermediates are prevalent in oxygen rich systems, while the blue coloured paths are prevalent in oxygen deficient systems. Adapted with permission from Sansotera *et al.* [149]. Copyright (2015) Elsevier.

In a study to evaluate the effect of oxygen on the degradation mechanism of PFOA, Sansotera *et al.* [149] established that apart from the stepwise degradation of PFOA by the elimination of CF_2_, another oxidation mechanism involving β-scission elimination of COF_2_ was involved in the degradation process as shown in Figure 12. The study also reported that while the stepwise CF_2_ elimination mechanism dominates in O_2_-deficient system, the β-scissions mechanism is dominant in O_2_-rich systems. In the β-scissions mechanism PFCAs are not generated as intermediates in the system.

### 5.2 Advanced reduction processes

**Figure 13:**
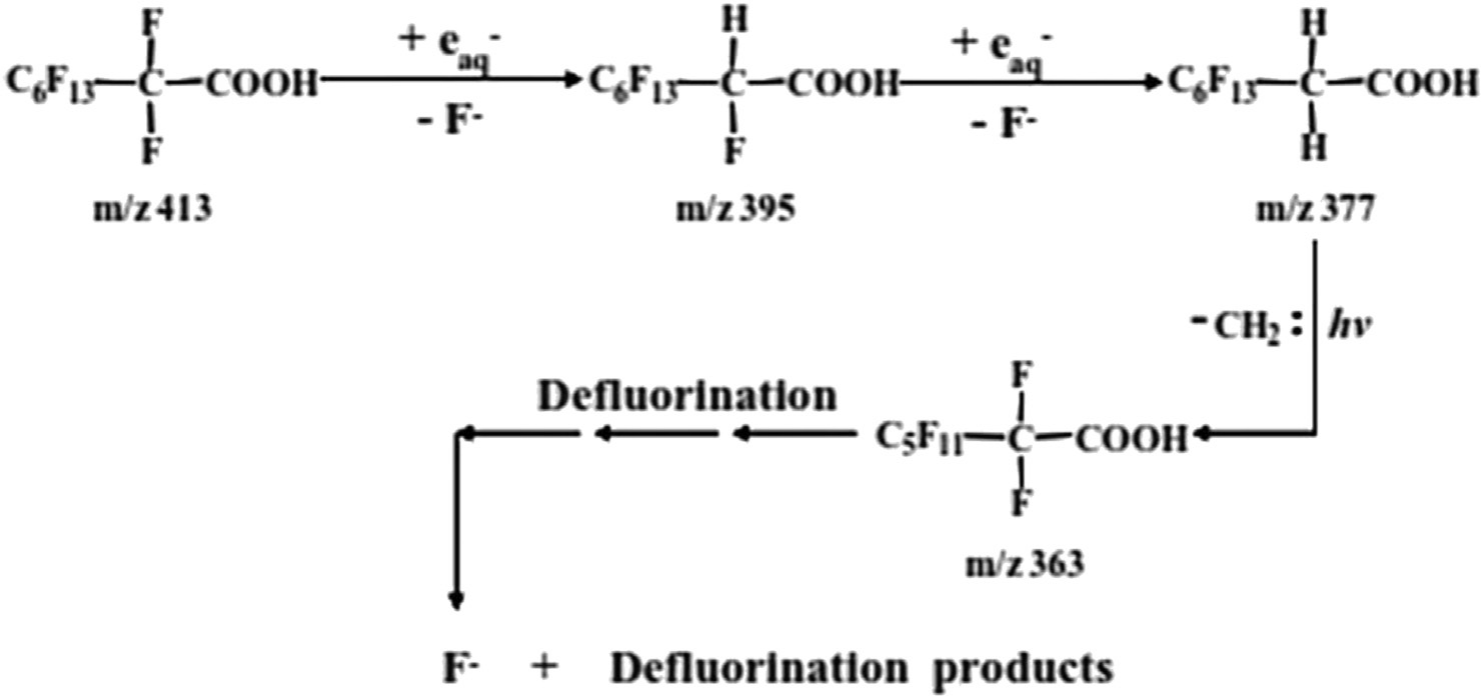
Photodegradation mechanism of PFOA. Reprinted with permission from Song *et al.* [151]. Copyright (2013) Elsevier.

The reductive degradation of PFAS are based mainly on hydrated electrons, due to their strong reducing power. Unlike oxidative degradation, ARPs are initiated by the cleavage of the α-C-F bond by the hydrated electrons because the reduction potential of the C-F bond (E˚ < -2.7 V) is lower than that of hydrated electron (E˚ = -2.9 V), which makes reduction relatively easier [130, 150]. This α-carbon attack continues step wisely to produce defluorinated carboxylic acids as shown in Figure 13 [150]. Different routes have been proposed for the hydrated electron's attack on the PFAS molecule.

The reductive degradation of PFASs is influenced by some factors, such as chemical characteristics of the C-F bond, stability of anion radical, redox potential, ionic head group, ^__^(CF_2_)_n_^__^ chain length, electron density and the strength of electron donating reagent [130, 152, 153, 154].

Bentel *et al.* [155] proposed two pathways for the reaction between hydrated electrons and PFCAs (C_n_F_2n+1_COO^−^). The first pathway involves sequential H/F exchange at the α-position, which yields C_n-1_F_2n-1_CH_2_COO^−^, with the possibility of additional C-F bond cleavage at middle positions for long chain PFCAs. The second pathway yielded shorter chain PFCAs through decarboxylation mechanism to produce unstable perfluorinated alcohol, which subsequently undergoes HF elimination to form acyl fluoride. The acyl fluoride then undergoes hydrolysis, leading to fluoride ion elimination to form shorter chain PFCA (C_n-1_F_2n-1_COO^−^).

**Figure 14:**
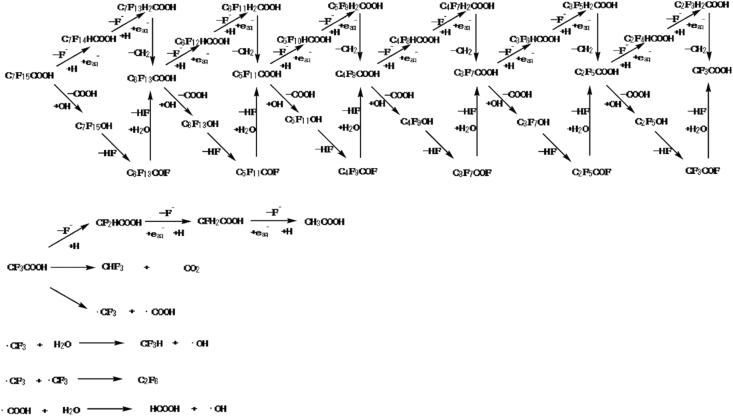
Mechanism of reductive degradation of PFOA. Reprinted wih permission from Qu *et al.* [142]. Copyright (2010) Elsevier.

Two reaction pathways for the degradation of PFOA (Figure 14), which include nucleophilic attack of hydrated electrons leading to reductive cleavage of C-F bonds, yielding defluorinated intermediates has also been proposed by Qu *et al.* [142]. Simultaneously, direct photolysis of C-C bonds occurred, resulting in the cleavage of the C-COOH bond, which yielded perfluoroalkyl group intermediates.

**Figure 15:**
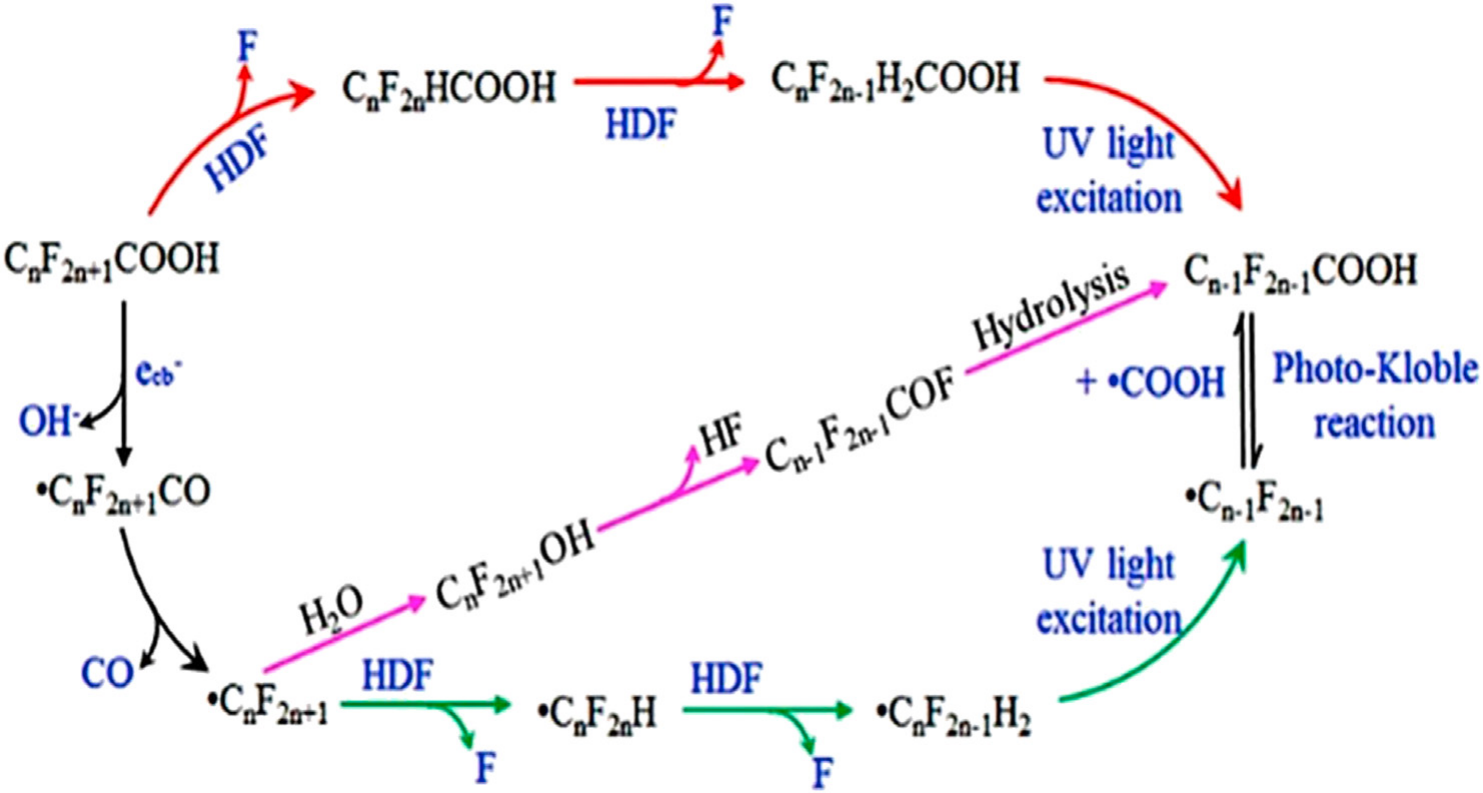
Mechanism of degradation of PFOA via the hydrodefluorination mechanism. Reprinted with permission from Huang *et al*. [156]. Copyright (2016) American Chemical society.

Recently, Huang *et al.* [156], proposed for the first time a hydrodefluorination mechanism to PFOA degradation by a SiC/graphene catalyst (Figure 15). The mechanism was based on the replacement of fluorine atoms by H atoms by nucleophilic substitution through Si-H/C-F redistribution. Si-H bonds were generated by UV light excitation, and the hydrogen atom exchanged for F atoms in the α-position to yield C_n_F_2n_HCOOH. The C_n_F_2n_HCOOH then went through a carbene (CH_2_) elimination under UV irradiation to form C_n-1_F_2n-1_COOH. Also, perfluoralkyl radical C_7_F^•^_15_ was formed by the reaction between photogenerated $e_{cb}^{-}$ with PFOA, which could then either undergo the HF elimination process or undergo the hydrodefluorination reaction.

## 6 Comparative analysis of photon enhanced processes

The comparative analysis of the photon-based processes for the degradation of PFAS in terms of their stage of development, relative cost, PFOA and PFOS removal efficiencies, materials and energy consumption is presented in Table 4.

### 6.1 Stage of development

The development stage of a treatment technology is based on the extent of application of the technology in different settings. The Environmental protection agency (EPA) [157] have established five categories of developmental stages for water treatment technologies: research, emerging, innovative, established, and adaptive use. Technologies that are currently in their developmental stage and have only been tested at bench scale are categorized as research, while technology that have undergone pilot or demonstration scale testing or have been implemented at full scale for less than 1 year are categorized as emerging. Innovative and established technologies are processes that have been implemented at full scale for less than five years and more than five years respectively. Adaptive use technologies are processes that have been established for years but have undergone modification which may have led to an emerging technology or may be adapted to achieve an alternative treatment objective. Table 5 shows the developmental stages of the photo enhanced process discussed in this review. Based on the mentioned criteria photolysis, photochemical and photocatalytic processes are classified as emerging technologies, while advanced reduction processes are still at the research stage based on the availability of pilot scale reports for each process [62, 158, 159].

**Table 5:** Comparative analysis of photon-based processes with other destructive technologies for PFAS degradation.

Technology		Development Stage	Energy (KWh/m^3^)	Relative cost ($)	Removal efficiency (%)	Average time required to 90% degradation (min)
AOP	Photolysis	Emerging	99.2	13.89	82	216
	Photochemical	Research	863.8	120.93	99	460
	Photocatalysis	Emerging	2106	294.84	89	705
ARP		Research	166.1	23.25	91.4	408

### 6.2 Energy consumption

In choosing between UV-based processes, one of the major factors to be considered is the energy consumption of the process [160]. The energy consumed in a process is measured by the electrical energy per order (EEO), which is defined as the electrical energy (KW/h) required to achieve 90% degradation of contaminants in 1 m^3^ stream. The E_EO_ could be obtained via the mathematical equation:

13 $$E_{EO}=\;\frac{P\;\times t\;\times 1000}{V\;\times 60\;\times log\frac{c_{i}}{c_{f}}}$$

Where P represents the systems power rating (in KW), t is the time required to achieve 90% degradation (in minutes), V is the volume of contaminant solution, the initial and final concentration of the contaminants are represented by C_i_ and C_f_ respectively [161]. The time required to achieve 90% degradation can be obtained from the reaction rate constant K (in mins) using the equation:

14 $$t_{0.9}=\;\frac{2.3035851}{k}$$

Using Eqs. (13) and (14), the energy consumption for the photolytic, photochemical, photocatalytic and photoreduction processes was calculated and are shown in Table 5 and it is observed that photolysis is the least energy consuming process, while photocatalytic process is the most energy consuming process. When compared to the energy consumption of other processes for PFAS degradation as presented by Nzeribe *et al.* [162], the energy consumption of photolysis compares favourably with that of plasma technology which was reported to have the lowest energy consumption when matched with activated persulfate, electrochemical oxidation and ultrasound technologies.

The average cost of a photo-enhanced process can be obtained by summing the average energy cost with the cost of the chemicals employed in the system. The energy cost for each system was obtained by multiplying the energy consumption of each process with the average energy cost worldwide, which in 2019 was $0.14 per kilowatt hour [163] (https://www.globalpetrolprices.com/electricity_prices). As shown by Nzeribe *et al.* [162], the contribution of chemical cost to the overall cost of most degradation processes was relatively insignificant, when compared to the energy cost. Also, for comprehensive cost analysis of this processes, there is the need for appropriate estimation of the cost of the materials required for reactors for the process. For instance, photochemical and advanced reduction degradation process, may require reactors made from corrosion resistant materials, which may also increase the overall cost of the degradation process.

### 6.3 Degradation efficiency and reaction time

In terms of efficiency, based on the average efficiencies reported by research explored in this review it can be observed that photochemical process had the highest process efficiency, followed by the advanced reduction process. These two technologies have achieved efficiencies greater than 90%. While photocatalysis and photolysis have average efficiencies of 89 and 82% respectively. However, in terms of the average time required to achieve 90% degradation, photolysis showed the lowest required time of 216 min, followed by advanced reduction process which required 408 min. Photochemical and photocatalytic processes both required an average time of 460 and 705 min respectively for 90% degradation to be achieved. Though photochemical degradation process showed the highest degradation efficiency among photon-based processes, when compared to other technologies such as electrochemical oxidation and activated persulphate, it showed the lowest degradation efficiency as presented by Nzeribe *et al.* [162].

## 7 Conclusion

Although, several technologies have been explored for the degradation of PFASs in the environment and wastewater in particular, this review has focussed on the treatment methods that employed photon energy in the degradation process. Studied reports have shown that persulfate and iron based photochemical process are the most effective advanced oxidation processes. The effectiveness has been ascribed to the stability and reactivity of $SO_{4}^{•\;-}$ and the ability of Fe^3+^ to complex with PFOA, which decomposes under UV-light. Among the semiconductor photocatalysts that have been explored, In_2_O_3_ showed the highest activity even under ambient reaction conditions. Advanced photoreduction processes, which are very recent technologies have also shown interesting efficiency in the degradation of PFAS. The study of the mechanism of degradation showed step-wise chain reduction as the primary degradation route. While initiation of the degradation process is by C-CO_2_H or C-SO_2_H cleavage in AOPs, direct cleavage of the C-F bond is the initial stage in ARPs.

In terms of cost and time required for PFAS degradation, comparative analysis showed that direct photolysis is an economically feasible process. However, the efficiency of the process is lower when compared to those of other processes. In terms of degradation efficiency, photochemical processes had the highest value, but the process is not potentially favoured for scale-up process.

Despite the interesting results reported for degradation of PFAS by photo enhanced processes, the need for highly efficient materials at ambient reaction conditions still remain to be explored. In addition, there is the need to reduce the time needed in achieving significant degradation of PFAS by these processes.

## Declarations

### Author contribution statement

All authors listed have significantly contributed to the development and the writing of this article.

### Funding statement

The study was supported by the 10.13039/501100001321National Research Foundation (UID 109333 and UID 116338).

### Data availability statement

No data was used for the research described in the article.

### Declaration of interests statement

The authors declare no conflict of interest.

### Additional information

No additional information is available for this paper.
